# Bromotyrosine-Derived Metabolites from a Marine Sponge Inhibit *Pseudomonas aeruginosa* Biofilms

**DOI:** 10.3390/ijms241210204

**Published:** 2023-06-16

**Authors:** Tam M. T. Tran, Russell S. Addison, Rohan A. Davis, Bernd H. A. Rehm

**Affiliations:** 1 Centre for Cell Factories and Biopolymers, Griffith Institute for Drug Discovery, Griffith University, Nathan, QLD 4111, Australia; 2 Preclinical ADME/PK, Griffith Institute for Drug Discovery, Griffith University, Nathan, QLD 4111, Australia; 3 NatureBank, Griffith Institute for Drug Discovery, Griffith University, Nathan, QLD 4111, Australia; 4 School of Environment and Science, Griffith University, Nathan, QLD 4111, Australia; 5 Menzies Health Institute Queensland, Griffith University, Gold Coast, QLD 4222, Australia

**Keywords:** *Pseudomonas aeruginosa*, biofilms, ianthelliformisamines, sponge, natural product, alkaloid, bromotyrosine, ciprofloxacin, cytotoxicity, in vitro metabolism

## Abstract

*Pseudomonas aeruginosa* forms stable biofilms, providing a major barrier for multiple classes of antibiotics and severely impairing treatment of infected patients. The biofilm matrix of this Gram-negative bacterium is primarily composed of three major exopolysaccharides: alginate, Psl, and Pel. Here, we studied the antibiofilm properties of sponge-derived natural products ianthelliformisamines A–C and their combinations with clinically used antibiotics. Wild-type *P. aeruginosa* strain and its isogenic exopolysaccharide-deficient mutants were employed to determine the interference of the compounds with biofilm matrix components. We identified that ianthelliformisamines A and B worked synergistically with ciprofloxacin to kill planktonic and biofilm cells. Ianthelliformisamines A and B reduced the minimum inhibitory concentration (MIC) of ciprofloxacin to 1/3 and 1/4 MICs, respectively. In contrast, ianthelliformisamine C (MIC = 53.1 µg/mL) alone exhibited bactericidal effects dose-dependently on both free-living and biofilm populations of wild-type PAO1, PAO1Δ*pslA* (Psl deficient), PDO300 (alginate overproducing and mimicking clinical isolates), and PDO300Δ*alg8* (alginate deficient). Interestingly, the biofilm of the clinically relevant mucoid variant PDO300 was more susceptible to ianthelliformisamine C than strains with impaired polysaccharide synthesis. Ianthelliformisamines exhibited low cytotoxicity towards HEK293 cells in the resazurin viability assay. Mechanism of action studies showed that ianthelliformisamine C inhibited the efflux pump of *P. aeruginosa*. Metabolic stability analyses indicated that ianthelliformisamine C is stable and ianthelliformisamines A and B are rapidly degraded. Overall, these findings suggest that the ianthelliformisamine chemotype could be a promising candidate for the treatment of *P. aeruginosa* biofilms.

## 1. Introduction

*Pseudomonas aeruginosa* (*P. aeruginosa*) is an opportunistic Gram-negative bacterium that causes various severe infections in hospitalized patients, hosts with impaired immune systems caused by human immunodeficiency virus (HIV) and cancer, and individuals with cystic fibrosis (CF). This bacterium possesses a range of virulence factors and adaptation mechanisms such as the ability to form biofilms, which have enabled resistance to multiple classes of antibiotics, leading to the treatment of infected patients being ineffective [1,2]. Several reports have demonstrated that bacteria in biofilm can resist antimicrobials up to 1000 times compared to their planktonic counterparts [3,4,5]. Evidently, highly structured biofilms are often identified in people with chronic infections (e.g., lung, wound, and rhinosinusitis) [6]. Therefore, much effort has been made towards the development of antibiofilm agents targeting these components and/or combination therapies with existing antibiotics to disarm and eventually eradicate this Gram-negative bacterium. 

Polysaccharides, extracellular DNA (eDNA), proteins, and lipids are the typical components of the *P. aeruginosa* biofilm matrix. The three exopolysaccharides (Psl, Pel, and alginate) were found to play critical roles in surface attachment and the formation and development of biofilms [7]. Psl, a neutral pentasaccharide composed of L-rhamnose, D-glucose, and D-mannose, is essential for cell attachment during the initial stage of biofilm formation [8,9,10]. Pel is a cationic polymer rich in *N*-acetyl-D-glucosamine and *N*-acetyl-D-galactosamine. It is necessary for surface adhesion, biofilm integrity, and the formation of pellicle biofilm at the air–liquid interface [11,12]. Alginate is an anionic acetylated polysaccharide polymer made of mannuronic acid and guluronic acid residues. Unlike Psl and Pel, alginate, substantially produced by the mucoid *P. aeruginosa* and typically isolated from the lung of CF patients, is a hallmark of chronic infections. This polysaccharide is involved in biofilm maturation and confers tolerance to antimicrobial treatments [13,14].

Natural products have been invaluable sources of medicinal agents for millennia. Indeed, their uses have been documented throughout history for the treatment of ailments including inflammation [15], cancer, and cardiovascular [16], neurological [17], and infectious diseases [18,19]. Many molecules from plants, marine organisms, and microorganisms have been shown to possess antibiofilm attributes such as organosulfur (garlic extracts) [20,21], brominated furanones and their analogs (red alga *Delisea pulchra*) [22,23], and promysalin (*Pseudomonas putida* RW10S1) [24,25]. Previously, ianthelliformisamines A–C were isolated from the Australian marine sponge *Suberea ianthelliformis* and several synthetic analogs were shown to have antibacterial activity against *P. aeruginosa* and to enhance the efficacy of doxycycline and chloramphenicol [26,27]. The anti-biofilm properties of ianthelliformisamines have not been investigated. In the present study, we investigated the effect of these compounds against *P. aeruginosa* biofilms. The synergistic interactions between ianthelliformisamines and clinically used antibiotics were determined. Furthermore, the interference of these molecules with different components of the biofilm matrix and the mode of action were studied. We further assessed the metabolic stability and cytotoxicity of ianthelliformisamines to evaluate their utility for treatment of *P. aeruginosa* infections.

## 2. Results

### 2.1. Minimum Inhibitory Concentrations of Ianthelliformisamines and Antibiotics

**Figure 1:**
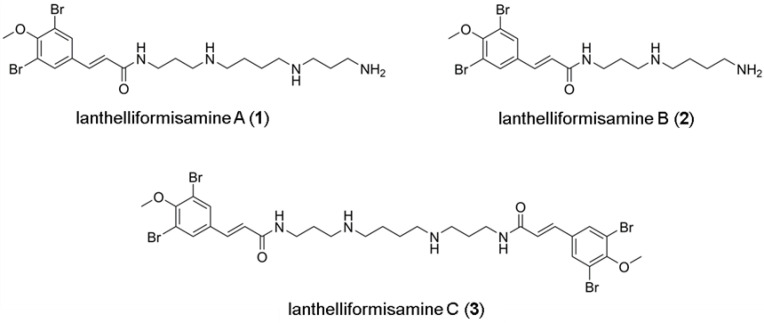
Chemical structures of ianthelliformisamines A–C.

Here, we investigated the anti-biofilm activity of ianthelliformisamines A–C (defined as compounds **1**–**3**) (Figure 1), which were previously shown to exhibit anti-bacterial activity against planktonic *P. aeruginosa*. Minimum inhibitory concentrations (MICs) of clinically used antimicrobials and compounds **1**–**3** were initially assessed against planktonic *P. aeruginosa* PAO1. The results revealed that MIC of ciprofloxacin, tobramycin, and meropenem against free-living PAO1 were 0.25 µg/mL, 2 µg/mL, and 0.375 µg/mL, respectively. An MIC of **3** was observed at 53.1 µg/mL. Compounds **1** and **2** showed no antimicrobial activity against *P. aeruginosa* PAO1 up to the highest concentrations applied in the present study.

### 2.2. Ianthelliformisamines A–C (***1**–**3***) Synergistically Interact with Ciprofloxacin to Inhibit Biofilm Formation

**Figure 2:**
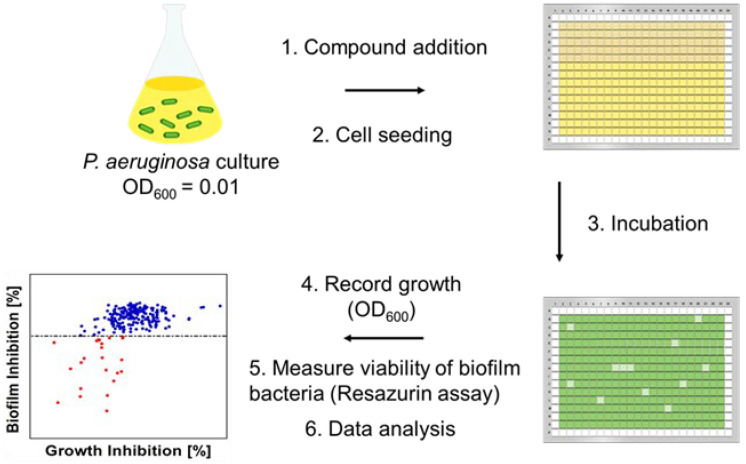
Biofilm assays to identify compounds that prevent biofilm formation. Biofilm inhibition assay. *P. aeruginosa* cultures were seeded into 384-well plates after dispensing compounds and antibiotics. The plates were incubated for 24 h at 37 °C followed by recording bacterial growth (OD_600_) and measuring the viability of biofilm bacteria using resazurin.

Initially, we investigated the antibiofilm activity of **1**–**3** against wild-type *P. aeruginosa* PAO1 using the biofilm inhibition assay (Figure 2). Compounds **1** and **2** did not yield any antimicrobial activity and/or prevented biofilm formation, whereas ianthelliformisamine **3** induced potent killing of free-living and biofilm bacteria of up to 30% and 60%, respectively. The activity of these compounds against existing biofilms of *P. aeruginosa* was also examined. No killing and/or dispersing effects on pre-formed biofilms were detected.

**Figure 3:**
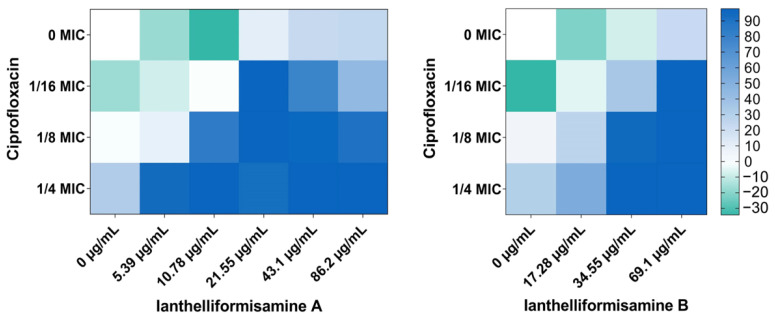
Ianthelliformisamine A and ianthelliformisamine B synergized with ciprofloxacin (CIP). Heat plots showing growth inhibition of planktonic PAO1 in the presence of compound and ciprofloxacin. Percent of growth inhibition is illustrated with different colors, where green represents growth stimulation and dark blue 100% inhibition.

Since these molecules were previously reported as antibiotic enhancers against planktonic PAO1, we then carried out a checkerboard assay to evaluate the potency of these compounds in the presence of other antibiotics including ciprofloxacin, tobramycin, and meropenem, which are used clinically either alone or in combination to treat *P. aeruginosa* infections. However, the current effectiveness of these agents is challenged by the rapid adaptation of the organism, due to an arsenal of virulence factors and biofilm architecture, resulting in an increasing prevalence of resistance in this Gram-negative bacterium. We reasoned that if the molecules could weaken the bacterial pathogenicity to some extent by attenuating virulence properties and impeding the capability of forming a biofilm, the antibiotics would be able to kill the bacteria at lower doses, thus alleviating the selection pressure-caused tolerance. The checkerboard assay revealed that **1** and **2** worked synergistically with ciprofloxacin at concentrations ranging from 5.39 µg/mL to 86.2 µg/mL for **1** and 34.55 µg/mL to 69.1 µg/mL for **2**, resulting in a 4–16-fold MIC reduction against wild-type PAO1 (Figure 3). Although the chemical structure of **3** is similar to compounds **1** and **2**, it exhibited limited synergy with ciprofloxacin.

**Figure 4:**
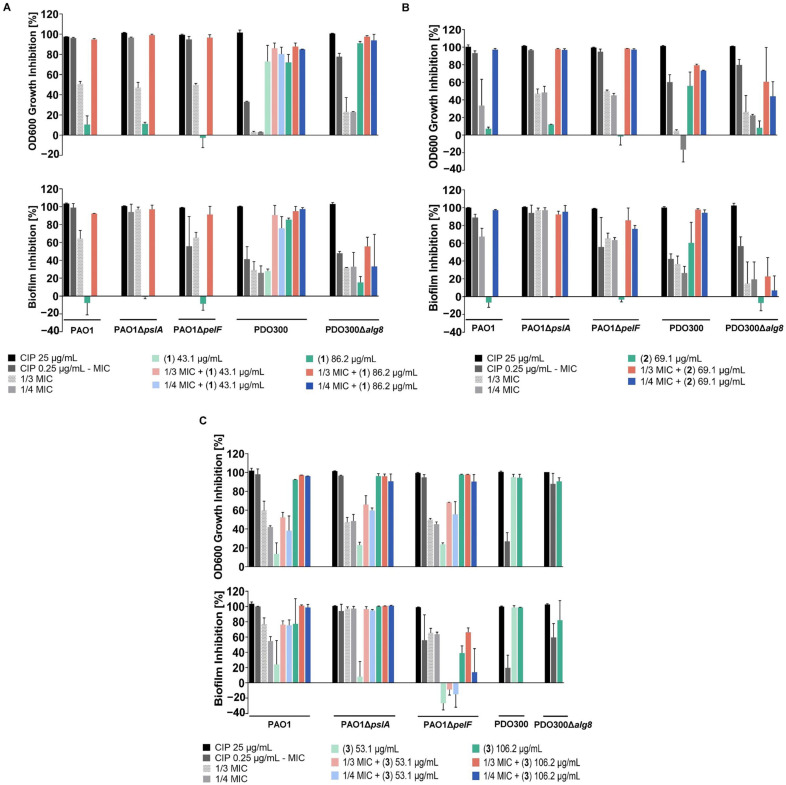
Ianthelliformisamines A–C (**1**–**3**) combined with ciprofloxacin resulted in decreased cell growth and biofilm formation. To assess interference with biofilm matrix components, wild-type and different isogenic mutant strains of *P. aeruginosa* PAO1, PAO1Δ*pslA*, PAO1Δ*pelF*, PDO300, and PDO300Δ*alg8* were treated with **1** (**A**), **2** (**B**), **3** (**C**), or ciprofloxacin, alone or in combination for 24 h. Cell growth (OD_600_) was monitored, and the resazurin assay was performed to determine the viability of biofilm bacteria. A culture with matched percentages of DMSO was used as the negative (untreated) controls. The experiment was conducted in triplicate. The results are percentage means and standard deviation (SD) of two independent experiments. Abbreviation: CIP, ciprofloxacin.

In order to target *P. aeruginosa* biofilms, synergistic combinations of **1** + ciprofloxacin and **2** + ciprofloxacin were selected for biofilm inhibition assays against wild-type and different isogenic mutant strains of *P. aeruginosa*. Higher concentrations of **1** and **2** in combination with ciprofloxacin were used in light of biofilm matrices serving as a major barrier, limiting concentrations of antibacterial compounds reaching cells within the biofilm. While these combinations showed no effects on established biofilms, they displayed different degrees of biofilm formation inhibition. Compound **1** + ciprofloxacin at 86.2 µg/mL and at 1/3 MIC (0.083 µg/mL), respectively, significantly disrupted growth and prevented biofilm formation of PAO1 and isogenic mutant strain PAO1Δ*pelF* (>90%) compared to ciprofloxacin or **1** alone (Figure 4A). In contrast, ciprofloxacin alone at 1/3 MIC moderately inhibited the bacterial growth and completely prevented biofilm formation of Psl-deficient strain PAO1Δ*pslA*. Therefore, the observed activity of the combination was presumably attributed to a sublethal level of ciprofloxacin. Similar synergism between **1** and ciprofloxacin was observed for alginate-overproducing mutant PDO300, which was generated to mirror pathogenesis of clinical isolates from the lungs of CF patients. The combination exerted equivalent levels of effect against PDO300 at all tested concentrations. We expected that the constitutive alginate production of the mucoid strain PDO300 [28] would hinder the penetration of **1** and the alginate deficient *alg8*-knockout mutant PDO300Δ*alg8* would ease the access of **1**. Unexpectedly, **1** acted alone on PDO300 growth at 43.1 µg/mL and 86.2 µg/mL (>70%) whereas only **1** at 86.2 µg/mL could reduce biofilm formation (~80%). Ciprofloxacin at 1/3 and 1/4 MICs only synergized with **1** at 43.1 µg/mL to improve their uptake into biofilms (from 20% to approximately 100%). This compound had minimal to moderate activity against PDO300Δ*alg8* biofilm bacteria (<60%), albeit showing the significant death of its planktonic counterparts, as indicated by the results of the biofilm assay (Figure 4A).

Compound **2** required a lower dose of ciprofloxacin to generate comparable effects with a combination of **1** and ciprofloxacin. Compound **2** (69.1 µg/mL) combined with 1/4 MIC (0.0625 µg/mL) ciprofloxacin caused growth inhibition and antibiofilm activity against PAO1 and PAO1Δ*pelF* (Figure 4B). Nonetheless, the addition of 1/3 and 1/4 MIC improved the suppression of PAO1Δ*pelF* biofilm by 20% and 13%, respectively. Again, ciprofloxacin alone at 1/3 and 1/4 MIC modestly reduced bacterial growth (>40%) but markedly suppressed biofilm formation of PAO1Δ*pslA* (~100%), while **2** alone produced marginal effects, indicating that there was no synergistic interaction between them. Exposure to **2** led to a blockage of the cellular progression of PDO300 (>50%), which appeared to be advanced in the presence of ciprofloxacin at 1/3 and 1/4 MICs (>70%) (Figure 4B). Additionally, the **2**/ciprofloxacin combination boosted the inhibition of PDO300 biofilms by nearly 40%. While survival of PDO300Δ*alg8* was compromised by 40% and 60% when treated with **2** + ciprofloxacin at 1/4 and 1/3 MIC, respectively, its biofilms were much less susceptible, suggesting that either diffusion through the secreted alginate was impaired or alginate captured the compounds.

Compound **3** at 53.1 µg/mL had negligible synergistic interactions with ciprofloxacin against planktonic forms as well as biofilms of PAO1, PAO1Δ*pslA*, and PAO1Δ*pelF* (Figure 4C). It, however, independently inhibited planktonic growth of the above strains at 106.2 µg/mL, demonstrating its antibacterial property. Additionally, biofilms of PAO1 and PAO1Δ*pslA* were significantly reduced by **3** alone at 106.2 µg/mL, up to 70% and 100%, respectively. In contrast, PAO1Δ*pelF* biofilms exhibited a reduced response to **3** (~40%) as opposed to ciprofloxacin alone (~60%) and the combinations (~60%). An MIC of **3** against PAO1 was observed at 53.1 µg/mL, instead of 106.2 µg/mL, in the MIC assay. Intriguingly, **3** (106.2 µg/mL) antagonized ciprofloxacin (1/4 MIC), which is evident by the decreased effectiveness against biofilm of PAO1Δ*pelF*, while the observed effects on planktonic cells were fully assigned to **3** in the presence of both compounds. The inability of PAO1Δ*pslA* to generate Psl may result in profound biofilm inhibition (~100%) when **3** (106.2 µg/mL) and sub-lethal doses of ciprofloxacin were applied. These concentrations of ciprofloxacin, on the other hand, partly affected survival of suspended cells (~50%). In addition, complete elimination of biofilm formation suggested that surviving cells remained in their planktonic forms after 24 h ciprofloxacin exposure. Unlike the sub-MIC of ciprofloxacin, **3** at 106.2 µg/mL completely killed the planktonic and biofilm populations of PAO1Δ*pslA*. Notably, the molecule **3** targeted both mutant strains PDO300 and PDO300Δ*alg8* at various degrees (Figure 4C). Free-living cells of both strains were eradicated, but the remaining survivors of PDO300Δ*alg8* were still able to form biofilms. Biofilm viability was severely reduced for PDO300 (~100%), albeit to a lesser extent for PDO300Δ*alg8* (80%).

Overall, the data suggested that ianthelliformisamines A–C (**1**–**3**) had the potential to function solely or to interact synergistically with the antibiotic ciprofloxacin against planktonic and biofilms of wild-type *P. aeruginosa* and an array of its isogenic mutants. Compounds **1** and **2** worked in combination with ciprofloxacin to compromise cell growth and biofilm adherence of PAO1 and PAO1Δ*pelF*. Only combinations of **2** and ciprofloxacin could effectively alter the biofilm formation of PDO300. Compound **3** alone targeted bacterial viability of PAO1, PAO1Δ*pslA*, PAO1Δ*pelF,* PDO300, and PDO300Δ*alg8*. Biofilms of these strains, apart from PAO1Δ*pelF* biofilms, were significantly inhibited by **3**. The absence of Psl considerably impaired the ability of PAO1Δ*pslA* to form biofilms when sub-inhibitory of ciprofloxacin or **3** at 106.2 µg/mL was present. In the current experiments, PAO1Δ*pelF* biofilms were the least affected by compound **3**. Furthermore, biofilms of PDO300Δ*alg8* were less susceptible to the compounds, ciprofloxacin, and the combinations compared to those of PDO300.

### 2.3. Influence of Ianthelliformisamines A–C (***1**–**3***) on the Outer Membrane Integrity

**Figure 5:**
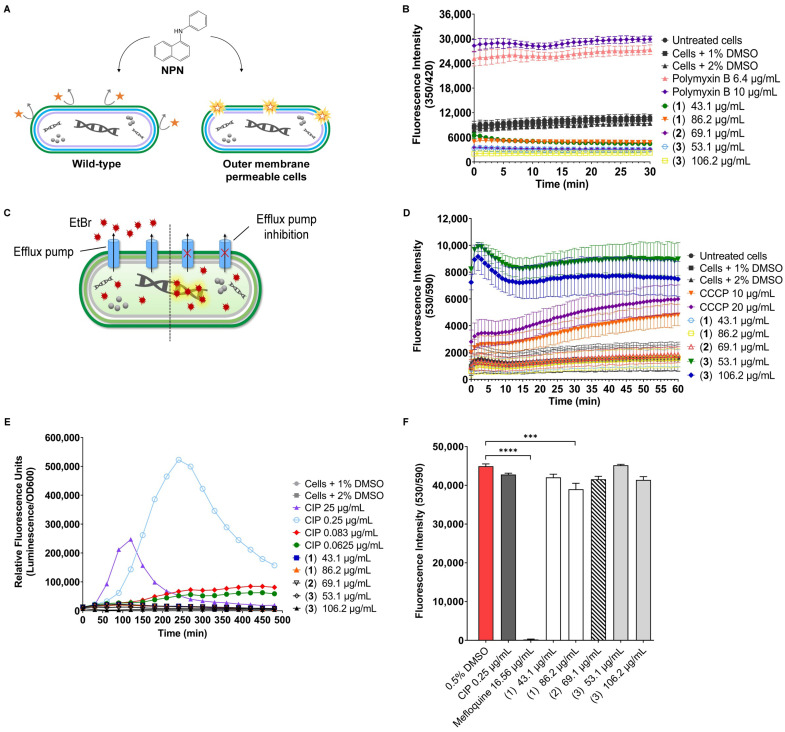
(**A**) The NPN dye is a lipophilic molecule that is used to determine permeability changes in bacteria. Such molecules are weakly fluorescent in an aqueous environment and impermeable to the cell with an intact outer membrane. However, once the membrane is damaged, NPN penetrates the cells and binds to the phospholipid layer, giving rise to pronounced fluorescence. (**B**) Wild-type PAO1 cells were treated with 100 µM CCCP prior to exposure to test compounds at different concentrations. The permeability changes in the outer membrane were assessed by monitoring the fluorescence of NPN for 30 min. Cells treated with DMSO solvent were included as the negative control. Polymyxin B was used as a positive control. (**C**) Ethidium bromide (EtBr) is a common substrate of the resistance-nodulation-cell division (RND) pump, which is used to measure the amount of intracellular accumulation, as it only fluoresces when bound to DNA. (**D**) The accumulation of EtBr in the presence of CCCP and test compounds was monitored for 60 min. Cells treated with DMSO solvent were included as the negative control. CCCP was used as a positive control. (**E**) Test compounds do not inhibit DNA synthesis. The reporter strain MDM-623 harboring the promoter region from *PA0614* fused to *P. luminescens luxCDABE* operon, a promoter-luciferase reporter gene, constructed to respond to DNA damage in general. The optical density OD_600_ and the kinetics of luminescence of the reporter strain were monitored for 8 h. Cells treated with DMSO solvent were included as the negative control, while ciprofloxacin at different concentrations was used as a positive control. (**F**) HEK293 resazurin viability assay. HEK293 cells were incubated with test compounds to evaluate cytotoxicity by resazurin assay. After 24 h of incubation, resazurin was dispensed into the wells, and the plate was further incubated for 5 h at 37 °C. Cells treated with DMSO solvent were included as the negative control. Mefloquine was used as a positive control. All experiments were performed in triplicate and repeated twice. The data are the means and SDs of two independent experiments. A non-parametric one-way ANOVA followed by Dunnett’s multiple comparison post-hoc test was carried out to determine statistical significance between each treatment and the negative control (0.5% DMSO).; ***, *p* < 0.001; ****, *p* < 0.0001. Abbreviation: CIP, ciprofloxacin.

The effect of ianthelliformisamines on the permeability of the outer membrane was determined using an NPN uptake assay. NPN (1-*N*-phenylnapthylamine), a hydrophobic fluorescent probe, is excluded from the intact outer membranes of Gram-negative bacteria such as *P. aeruginosa*. It intensely fluoresces once it gains access to the phospholipid layer of membrane-compromised cells (Figure 5A) [29]. The experiments were conducted with different concentrations of ianthelliformisamines and sub-inhibitory doses of ciprofloxacin. We observed that cells treated with polymyxin B at 6.4 µg/mL and 10 µg/mL, an outer membrane-active compound, exhibited prominent fluorescence compared to DMSO solvent-treated cells and untreated cells (Figure 5B). The level of fluorescence intensity in bacteria challenged with test compounds, even at highest testing concentrations, resembled that in cells treated with DMSO. The results indicated that ianthelliformisamines are not membrane permeabilizers.

### 2.4. Efflux Assay

We performed the ethidium bromide (EtBr) efflux assay to assess the ability of test compounds to inhibit efflux pumps of *P. aeruginosa.* The DNA-intercalating dye EtBr is a common substrate of the resistance-nodulation-cell division (RND) family. The dye only fluoresces when bound to DNA in the cytoplasm. The disruption of efflux pumps such as the RND pump AcrB enhances the accumulation of the substrate within the cells over time until it reaches a steady state [30]. (Figure 5C). Cells treated with compounds **1** and **2** produced comparable EtBr accumulation with untreated bacteria and the DMSO-treated population. It was shown that only compound **3** at 53.1 µg/mL and 106.2 µg/mL inhibited the efflux pump. The level of intracellular EtBr accumulation by **3** was greater than that mediated by reference compound CCCP, a chemical efflux pump inhibitor that blocks the gradient of protons across the membrane [31]. The fluorescence intensity induced by **3** at 53.1 µg/mL (MIC) remained higher than at 106.2 µg/mL (2 × MIC) over the time course, suggesting dose-dependent inhibition (Figure 5D). 

### 2.5. Do Ianthelliformisamines A–C (***1**–**3***) Share the Same Mode of Action with Ciprofloxacin?

Ciprofloxacin, a fluroquinolone antibiotic, has been extensively utilized to treat various bacterial infections. It targets bacterial DNA topoisomerase and DNA gyrase, which produce breakage of double-stranded DNA, ultimately leading to cell death [32]. In the present study, compounds **1** and **2** did not enhance the activity of tobramycin (aminoglycoside) or meropenem (carbapenem) but exclusively worked with ciprofloxacin (Figure 3). In the effort of identifying the mechanisms of action of test compounds, we employed a DNA damage reporter strain MDM-623, which was constructed based on the promoter-luciferase reporter gene. The increased luminescence readouts are indicative of *gyrase* inhibition-induced promoter upregulation through the *recA* pathway [33]. The luminescence signal was steadily enhanced in response to ciprofloxacin treatments (positive controls) at 1/4 MIC, 1/3 MIC, 1 × MIC, and 100 × MIC, equivalent to 0.0625, 0.083, 0.25, and 25 µg/mL, respectively, compared to DMSO treated cells (negative control). Nevertheless, there was no difference in the response of MDM-623 upon exposure to test compounds and the solvent (Figure 5E). The above results may imply that test compounds are unlikely to be DNA gyrase inhibitors and that their mechanism of action is unrelated to that of ciprofloxacin.

### 2.6. Effect of Ianthelliformisamines A–C (***1**–**3***) on HEK293 Cells

We sought to determine the cytotoxicity of the compounds using a resazurin viability assay. The compound treatments induced low toxicity to HEK293 cells up to the highest testing concentrations (Figure 5F), indicating their potential suitability for therapeutic use.

### 2.7. Fluorescence Microscopy Imaging Demonstrates Synergism between Ianthelliformisamines A–C (***1**–**3***) and Ciprofloxacin

**Figure 6:**
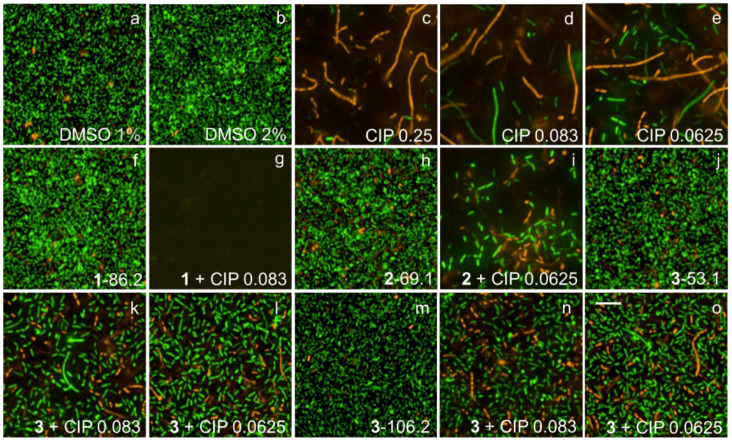
Representative fluorescence images of treated and untreated PAO1 biofilms. Cells treated with ciprofloxacin and test compounds **1**–**3** were stained with SYTO9 (green) and propidium iodide (red). Ciprofloxacin generated long, filamentous phenotypes as well as small, round spheroplasts. Biofilms challenged with **1** and **2** remained unaffected, while that with **3** was slightly reduced. Complete biofilm inhibition was observed with 1 + 1/3 MIC. Abbreviations: **1**–**3** (ianthelliformisamines A–C), CIP (ciprofloxacin). Cells treated with (**a**) DMSO 1%, (**b**) DMSO 2%, (**c**) ciprofloxacin at 1 × MIC, (**d**) ciprofloxacin at 1/3 MIC, (**e**) ciprofloxacin at 1/4 MIC, (**f**) **1** at 86.2 µg/mL, (**g**) **1** 86.2 µg/mL + CIP 1/3 MIC, (**h**) **2** at 69.1 µg/mL, (**i**) **2** 69.1 µg/mL + CIP 1/4 MIC, (**j**) **3** at 53.15 µg/mL, (**k**) **3** 53.1 µg/mL + CIP 1/3 MIC, (**l**) **3** 53.1 µg/mL + CIP 1/4 MIC, (**m**) **3** at 106.2 µg/mL, (**n**) **3** 106.2 µg/mL + CIP 1/3 MIC, and (**o**) **3** 106.2 µg/mL + CIP 1/4 MIC. Scale bar 10 µM.

**Figure 7:**
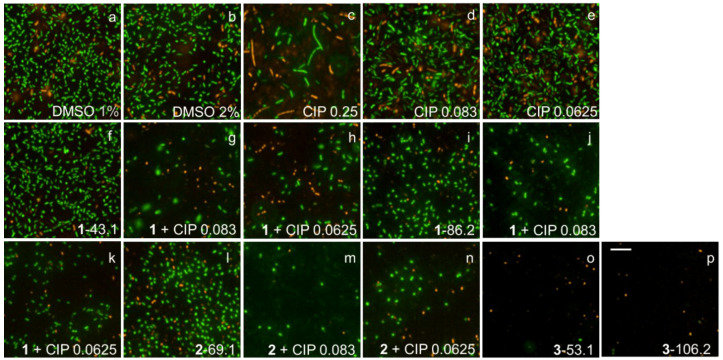
Representative fluorescence images of treated and untreated PDO300 biofilms. Cells treated with ciprofloxacin and test compounds **1**–**3** were stained with SYTO9 (green) and propidium iodide (red). Ciprofloxacin generated long, filamentous phenotypes as well as small, round spheroplasts. Biofilms challenged with **1** and **2** slightly reduced, while those challenged with **3** were completely eradicated. Abbreviations: **1**–**3** (ianthelliformisamines A–C), CIP (ciprofloxacin). Cells treated with (**a**) DMSO 1%, (**b**) DMSO 2%, (**c**) ciprofloxacin at 1 × MIC, (**d**) ciprofloxacin at 1/3 MIC, (**e**) ciprofloxacin at 1/4 MIC, (**f**) **1** at 43.1 µg/mL, (**g**) **1** 43.1 µg/mL + CIP 1/3 MIC, (**h**) **1** 43.1 µg/mL + CIP 1/4 MIC, (**i**) **1** at 86.2 µg/mL, (**j**) **1** 86.2 µg/mL + CIP 1/3 MIC, (**k**) **1** 86.2 µg/mL + CIP 1/4 MIC, (**l**) **2** at 69.1 µg/mL, (**m**) **2** 69.1 µg/mL + CIP 1/3 MIC, (**n**) **2** 69.1 µg/mL + CIP 1/4 MIC, (**o**) **3** at 53.1 µg/mL, and (**p**) **3** at 106.2 µg/mL. Scale bar 10 µM.

**Figure 8:**
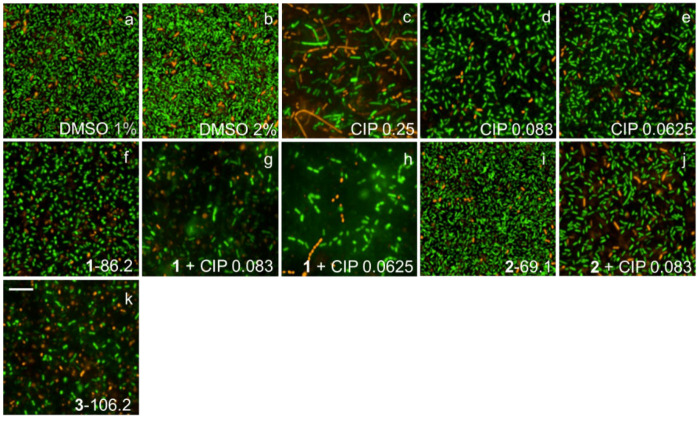
Representative fluorescence images of treated and untreated PDO300Δ*alg8* biofilms. Cells treated with ciprofloxacin and test compounds **1**–**3** were stained with SYTO9 (green) and propidium iodide (red). Ciprofloxacin generated long, filamentous phenotypes as well as small, round spheroplasts. Biofilms challenged with **1**–**3** were inhibited at different levels. Abbreviations: **1**–**3** (ianthelliformisamines A–C), CIP (ciprofloxacin). Cells treated with (**a**) DMSO 1%, (**b**) DMSO 2%, (**c**) ciprofloxacin at 1 × MIC, (**d**) ciprofloxacin at 1/3 MIC, (**e**) ciprofloxacin at 1/4 MIC, (**f**) **1** at 86.2 µg/mL, (**g**) **1** 86.2 µg/mL + CIP 1/3 MIC, (**h**) **1** 86.2 µg/mL + CIP 1/4 MIC, (**i**) **2** at 69.1 µg/mL, (**j**) **2** 69.1 µg/mL + CIP 1/3 MIC, and (**k**) **3** at 106.2 µg/mL. Scale bar 10 µM.

**Figure 9:**
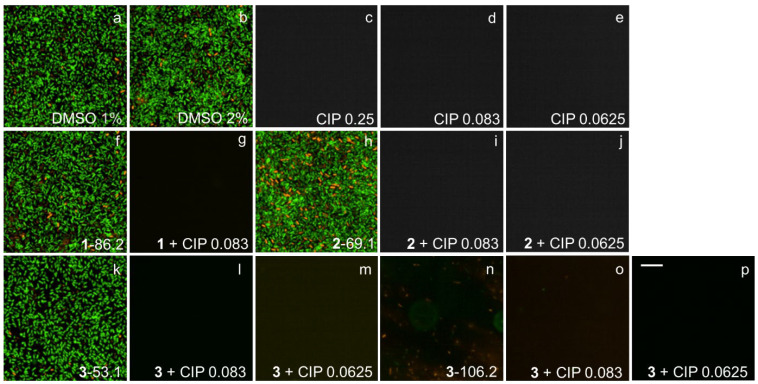
Representative fluorescence images of treated and untreated PAO1Δ*pslA* biofilms. Cells treated with ciprofloxacin and test compounds **1**–**3** were stained with SYTO9 (green) and propidium iodide (red). PAO1Δ*pslA* was unable to form biofilms in the presence of ciprofloxacin. Biofilms challenged with **1**–**3** remained unaffected. Abbreviations: **1**–**3** (ianthelliformisamines A–C), CIP (ciprofloxacin). Cells treated with (**a**) DMSO 1%, (**b**) DMSO 2%, (**c**) ciprofloxacin at 1 × MIC, (**d**) ciprofloxacin at 1/3 MIC, (**e**) ciprofloxacin at 1/4 MIC, (**f**) **1** at 86.2 µg/mL, (**g**) +**1** 86.2 µg/mL + CIP 1/3 MIC, (**h**) **2** at 69.1 µg/mL, (**i**) **2** 69.1 µg/mL + CIP 1/3 MIC, (**j**) **2** 69.1 µg/mL + CIP 1/4 MIC, (**k**) **3** at 53.1 µg/mL, (**l**) **3** 53.1 µg/mL + CIP 1/3 MIC, (**m**) **3** 53.1 µg/mL + CIP 1/4 MIC, (**n**) **3** at 106.2 µg/mL, (**o**) 3 106.2 + CIP 1/3 MIC µg/mL, and (**p**) **3** 106.2 + CIP 1/4 MIC µg/mL. Scale bar 10 µM.

**Figure 10:**
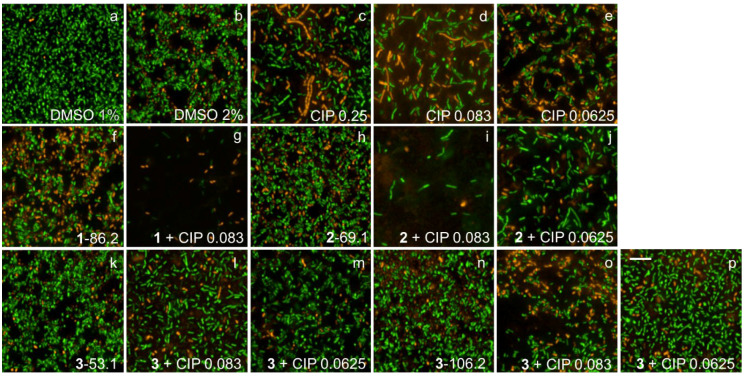
Representative fluorescence images of treated and untreated PAO1Δ*pelF* biofilms. Cells treated with ciprofloxacin and test compounds **1**–**3** were stained with SYTO9 (green) and propidium iodide (red). Ciprofloxacin generated long, filamentous phenotypes as well as small, round spheroplasts. Biofilms challenged with **1**–**3** remained unaffected. Abbreviations: **1**–**3** (ianthelliformisamines A–C), CIP (ciprofloxacin). Cells treated with (**a**) DMSO 1%, (**b**) DMSO 2%, (**c**) ciprofloxacin at 1 × MIC, (**d**) ciprofloxacin at 1/3 MIC, (**e**) ciprofloxacin at 1/4 MIC, (**f**) **1** at 86.2 µg/mL, (**g**) **1** 86.2 µg/mL + CIP 1/3 MIC, (**h**) **2** at 69.1 µg/mL, (**i**) **2** 69.1 µg/mL + CIP 1/3 MIC, (**j**) **2** 69.1 µg/mL + CIP 1/4 MIC, (**k**) **3** at 53.1 µg/mL, (**l**) **3** 53.1 µg/mL + CIP 1/3 MIC, (**m**) **3** 53.1 µg/mL + CIP 1/4 MIC, (**n**) **3** at 106.2 µg/mL, (**o**) **3** 106.2 µg/mL + CIP 1/3 MIC, and (**p**) **3** 106.2 µg/mL + CIP 1/4 MIC. Scale bar 10 µM.

Biofilms of wild-type PAO1 and isogenic biofilm matrix component mutants were treated with ianthelliformisamines or ciprofloxacin alone and the combinations. Their biofilms were simultaneously stained with SYTO9 (green) and propidium iodide (red). Cells with intact membranes were stained green, while cells with compromised membranes were stained red. Representative images of cells challenged with different treatments including DMSO, ianthelliformisamines, ciprofloxacin, and synergistic combinations are given in Figure 6, Figure 7, Figure 8, Figure 9 and Figure 10. Analysis of the images showed that ciprofloxacin treatment induced filamentous growth in dose-dependent and strain-specific manners. At 1 × MIC, ciprofloxacin produced reduced viability and long, filamentous phenotypes in all tested strains apart from PAO1Δ*pslA*. The phenotypes were also observed at sub-lethal doses in ciprofloxacin-treated PAO1 and PAO1Δ*pelF.* In addition, ciprofloxacin generated another small, round cell population, namely spheroplasts [34].

Treatment of PAO1 biofilms showed that compound **1** or compound **3** acted synergistically or antagonistically, respectively, with ciprofloxacin. Compound 2 had no effect. When treating biofilms formed by the alginate overproducer PDO300, compounds **1** and **2** each in combination with ciprofloxacin showed synergistic effects. Interestingly, compound **3** eradicated the PDO300 biofilm. All compounds had little effect on the PDO300Δ*alg8* biofilm, suggesting that presumably, enhanced Psl and/or Pel production in this mutant interfered with antibacterial activity. This is further corroborated by the sensitivity PAO1Δ*pslA* biofilms that were eradicated by ciprofloxacin alone or in combination with compounds **1** and **2**, while only compound **3** alone completely dispersed the biofilm. The PAO1Δ*pelF* biofilm was susceptible to the synergistic effects of compounds **1** and **2** each combined with ciprofloxacin, but compound **3** showed no effect with or without ciprofloxacin.

### 2.8. In Vitro Metabolism of Ianthelliformisamines A–C (***1**–**3***)

Metabolic degradation, in vivo half-life, is an important criterion for the applicability of potential drugs. Here, we used surrogate in vitro assays to study the metabolic stability of compounds **1**–**3**.

### 2.9. Positive Controls

A single positive control sample using verapamil as the control substance was run concurrently with the test samples in microsomes from all species. Verapamil was rapidly metabolized using the experimental conditions used for compounds **1**–**3**, with half-lives of approx. 6 min (mouse) and 16 min (human). Results for the positive control incubations are provided in the Supplementary Data.

### 2.10. Ianthelliformisamine A (***1***)

The results for the degradation of **1** (as % remaining vs. time) in mouse liver microsomes are provided in Table S1 and Figure S1A, while the graphical representation for LN-transformed data is shown in Figure S1B. In mouse microsomes, **1** exhibited essentially linear degradation over the 0–30-min period (mean half-life of degradation = 38.2 min), with a decrease in the rate of degradation evident between the 30- and 60-min timepoints. As the clearance of any compound should be determined to reflect that resulting from initial kinetics, the LN-transformed data and clearance estimations were calculated over the 0–30-min period in these microsomes.

In contrast, the negative control tube (no NRS added, therefore no CYP450 metabolism possible) displayed no degradation to **1** over the first 20 min of incubation, followed by a decrease between 20 and 60 min with a half-life of approximately 45 min. Any degradation in a negative control tube without added NRS may be due to non-NADPH-dependent enzymatic metabolism (i.e., non-CYP450 metabolism) such as carboxylesterases or to general instability in aqueous media [35]. Given the difference between the test and negative control samples over the first 20 min, it is reasonable to conclude that oxidative metabolism was still the dominant metabolic process occurring to **1** in these microsomes, but this non-oxidative degradation observed after the 20-min point should still be considered in the overall stability of this compound. Results for individual tubes for incubations of **1** in mouse liver microsomes are provided in the Supplementary Materials, while calculated stability parameters based on the NADPH-dependent degradation profiles obtained are provided in Table S2. In human microsomes, **1** demonstrated substantial instability, with no meaningful half-life or clearance data being able to be obtained in these microsomes. Indeed, the response to the test compound in the LC-MS/MS decreased to minor levels over the time taken to analyze the samples from a metabolism experiment (approx. 2.5 h). All samples were kept on ice following addition to the internal standard solution, and for **1**, the samples were only moved to the LC-MS/MS in small batches immediately prior to analysis. These investigations in human microsomes were repeated on separate occasions, but no meaningful data was obtained from either attempt. In contrast, the results obtained for the positive control (verapamil) conducted alongside the test compound worked well on all occasions, confirming the validity of the actual incubations conducted. In addition, the linearity of response for **1** over an appropriate concentration range using standards prepared in phosphate buffer (but no microsomal protein) was confirmed prior to the metabolism experiments, thereby confirming that the analytical conditions used were appropriate.

Based on the experimental half-life of 38.2 min, **1** showed moderate degradation in mouse liver microsomes. This result equates to a hepatic extraction of 0.48, which corresponds to an intermediate level of hepatic extraction in this species, based on the classification system proposed by Houston 1994 [36].

### 2.11. Ianthelliformisamine B (***2***)

The results for the degradation of **2** (as % remaining vs. time) in mouse and human liver microsomes are provided in Table S3 and Figure S2A, while graphical representations for LN-transformed data are shown in Figure S2B,C. For both species, the degradation of **2** was essentially linear over the 5–30-min time period, and LN-transformed data from this period was used for clearance determinations due to superior linearity. The rates of degradation of **2** observed in microsomes from both species were similar (mean ± SD = 42.8 ± 7.6 min [mouse] and 35.7 ± 2.1 min [human]), which in turn were similar to those observed for **1** in mouse liver microsomes (38.2 min).

It is noted that the results shown in Table S3 for the mouse microsomes and for the t = 5 min sample in human microsomes, when normalized to the t = 0 result, were >100%. This is most likely due to an artefactually low result being obtained for the t = 0 samples; the cause of this is not known. Given the good linearity obtained between 5 and 30 min, the data for calculation of clearance has been normalized to the t = 5 min result.

In both species, the negative control tubes (no NRS added) showed a slow level of degradation over the 30-min incubation period (half-life of degradation = 90.0 min [mouse] and 103.4 min [human]). As indicated above for **1**, any degradation observed in the negative control tube may occur due to non-NADPH-dependent enzymatic metabolism (i.e., non-CYP450 metabolism) such as carboxylesterases or to general instability in aqueous media [35], and it is again reasonable to conclude that oxidative metabolism was still the dominant metabolic process occurring to **2** in the microsomes. However, the slow non-oxidative degradations observed should still be considered in the overall stability of this compound.

Results for individual tubes for incubations of **2** in microsomes from both species are provided in the Supplementary Materials. Calculated stability parameters for **2**, based on the NADPH-dependent degradation profiles obtained, are provided in Table S4.

Based on the mean experimental half-lives of 42.8 and 35.7 min, **2** showed moderate degradation in both mouse and human liver microsomes. These results equate to hepatic extractions of 0.42 and 0.61 in mouse and human liver microsomes, respectively, which correspond to intermediate levels of hepatic extraction, based on the classification system proposed by Houston 1994 [36]. 

### 2.12. Ianthelliformisamine C (***3***)

Compound **3** was metabolically stable in incubations with both mouse and human microsomes under the conditions employed. No decrease in concentration was observed over the 0–60-min incubation period in human microsomes or for the 0–30-min period in mouse microsomes, with only a slight decrease in mean concentration in mouse liver microsomes between 30 and 60 min (mainly caused by a lower result for one of the triplicate tubes—see Supplementary Materials). Consequently, no calculations for half-life or hepatic extraction were possible for **3** in either species.

The results for **3** (as % remaining vs. time) in mouse and human liver microsomes are provided in Table S5 and Figure S3A, while graphical representations for LN-transformed data are shown in Figure S3B,C.

Generally, in the presence of human liver microsomes, **1** demonstrated substantial instability, with no meaningful half-life or clearance data being able to be obtained in these microsomes despite repeated attempts. For **2**, the mean (*n* = 3) degradation half-lives in mouse and human liver microsomes were 42.8 min and 35.7 min, respectively, over the 0–30-min incubation period. These equate to calculated in vivo hepatic extractions of 0.42 and 0.61, respectively. In contrast, **3** was metabolically stable in incubations using both mouse and human microsomes under the conditions employed. No decrease in concentration was observed over the 0–60 min incubation period; therefore, no calculation of half-life or hepatic extraction was possible.

## 3. Discussion

The rapidly increasing antibiotic resistance among bacterial pathogens has posed global health threats. Therefore, there is an urgent need for new antimicrobial compounds, especially for Gram-negative bacteria, as emphasized by the World Health Organization. One of the key defense mechanisms of bacteria is the formation of biofilms, which confer protection to embedded cells from antibiotic treatments and an escape from the host immune system, eventually leading to chronic infections. Several strategies such as combination therapies that combine two or more therapeutic agents have been proven to be efficient in addressing this increasingly widespread multi-drug resistance (MDR) and their biofilm-associated infections by targeting various molecular pathways in a synergistically or additive manner [37].

Previously, ianthelliformisamines A–C (Figure 1) and their derivatives have been reported as promising and broad-spectrum antimicrobials as well as antibiotic enhancers against *Enterobacter aerogenes*, *P. aeruginosa*, and *Klebsiella pneumoniae* MDR strains [26,27]. To our knowledge, the efficacy of **1**–**3** and their combinations with antibiotics against biofilms of *P. aeruginosa* has not been investigated. In the present study, we sought to determine the antibiofilm activities of these molecules individually and in combinations with common antibiotics used in clinical settings (Figure 2 and Figure 3).

In current experimental conditions, **1** and **2** alone exerted insignificant effects on free-living cells and biofilm formation of all the studied strains up to the highest tested concentrations (Figure 3). The results were in accordance with previous work, which reported that the MICs were 172.4 µg/mL for **1** and >138.2 µg/mL for **2**, which were two-fold greater than the currently studied doses [27]. MIC of **3** was previously determined at ~26.55 µg/mL [27], whereas it was 53.1 µg/mL in the current study (Figure 4). The variation could be due to the differences in experimental conditions, such as culture medium and initial inoculum. Interestingly, **3** at 106.2 µg/mL displayed potent activity on planktonic forms of all the strains, despite its high molecular weight (833.96 g/mol) as opposed to its analogues, suggesting its bactericidal properties. It raised the possibility that the outer membrane was permeabilized to allow the intake of such a large molecule. The cell envelope of Gram-negative bacteria is a complex structure consisting of an outer membrane, a peptidoglycan cell wall, and an inner membrane. The outer membrane contains channel-forming proteins, porins, which serve as a selective barrier allowing the influx of essential nutrients into the cells, extruding waste and defending against toxic chemicals. Generally, these porins allow passive diffusion of hydrophilic solutes with sizes up to 600 Daltons. *P. aeruginosa* possesses a low number of porins with a slow penetration rate, thus reducing the permeability of the outer membrane, further aiding resistance to antibiotics [38,39]. Nonetheless, the data of the NPN uptake assay indicated that none of the test compounds were membrane-active (Figure 5).

Furthermore, compound **3** impeded the biofilm formation of PAO1Δ*pslA*, PDO300, and PDO300Δ*alg8* (~100%) but to a lesser extent that of wild-type PAO1 (>70%) (Figure 4). It was shown that deletion of *pslA* resulted in impairment of initial surface attachment due to decreased level of signaling molecule c-di-GMP (bis-(3′-5′)-cyclic dimeric guanosine monophosphate) [40] and increased production of Pel in static biofilms; the latter was incapable of compensating for this deficiency, as corroborated by a reduction in biofilm volume [41]. It is worth mentioning that Pel-rich biofilms enhanced the tolerance to DNase digestion and charged antibiotics such as tobramycin [42]. Hence, the elevated level of Pel in the biofilms of PAO1Δ*pslA* did not confer any protection from the neutral antibiotic ciprofloxacin. Psl plays an essential role in initial adherence and functions as a protective layer for biofilm bacteria against antibiotics such as tobramycin and ciprofloxacin [43] and host immune responses like phagocytosis [44]. Moreover, it is commonly found in many cystic fibrosis (CF) isolates, and its combination with alginate triggered severe damage to surrounding host tissues via substantial production of extracellular reactive oxygen species (ROS) [44]. Collectively, these perhaps explain the susceptibility of PAO1Δ*pslA* biofilms to compound **3** and sublethal ciprofloxacin. They also suggest that Psl may be a promising target to improve biofilm eradication treatments [45]. Biofilms of PAO1Δ*pelF* were the least affected by **3** and its combinations compared to those of other studied strains. The only difference in biofilms of PAO1Δ*pelF* is the deficiency of Pel. It is unclear why the absence of Pel generated the tolerance to treatments. A conversion from nonmucoid to mucoid phenotype appears to be a crucial adaptative mechanism for chronic infections of CF lung [46]. The mutant PDO300 was constructed to mimic the adaptation in CF patient lung infected with *P. aeruginosa* [33]. It is interesting that molecule **3** effectively reduced the viability of suspended cells and biofilm formation of the mucoid variant PDO300, whose biofilms have been shown to have a high level of tolerance to tobramycin (up to 1000 times) compared to biofilms formed by nonmucoid PAO1 [47]. The overproduction of alginate in PDO300 due to defective *mucA22* allele hindered solid surface attachment [28] and retarded the growth rate. The presence of **3** may further impact the viability and impede its ability to form biofilms. Previous work reported that although PDO300Δ*alg8* formed flat biofilms, its biofilms were found to be more compact and contained more dead cells and extracellular DNA than other biofilms. Additionally, a lack of alginate led to increased production of Pel polysaccharide, which subsequently enhanced cell-to-cell interaction as well as the biofilm compactness [41], thus possibly making it less accessible to compound **3**. This might be the reason why the effect of **3** on PDO300Δ*alg8* biofilms was weaker than on PDO300. Of note, Pel-deficient biofilms (PAO1Δ*pelF*) were more resistant to **3** treatment (106.2 µg/mL) than alginate-deficient biofilms (PDO300Δ*alg8*) and mucoid biofilm (PDO300), in spite of similar planktonic killing.

To improve the activities of **1**–**3**, we carried out synergy assays. In our experiment, these compounds exclusively synergized with fluoroquinolone ciprofloxacin, a DNA synthesis inhibitor. Improvement of planktonic killing and antibiofilm activity in a strain-specific manner, though at different levels, following the treatment with combinations of **1** or **2** or **3** and ciprofloxacin were observed. This is in line with a recent study suggesting that ianthelliformisamines and its derivatives were antibiotic potentiators [27]. In addition to reduced outer membrane permeability, *P. aeruginosa* possesses an efficient efflux system, contributing to antibiotic resistance worldwide. Our next attempt was to explore the modes of action of these molecules. In efflux assays, only compound **3** was able to advance the intracellular EtBr accumulation. The fact that the fluorescence intensity was higher in cells treated with **3** than that in cells treated with CCCP, a known efflux pump inhibitor, indicated that **3** is an efflux pump inhibitor (Figure 5). Additionally, the interactions between ianthelliformisamines and ciprofloxacin were also studied. Synergy interactions occur when two molecules work on distinct signaling pathways that could be activated by the same signal. Additivity, on the other hand, happens when two molecules share the same mechanism of action [48]. Since ciprofloxacin induces cell filamentation and inhibits DNA synthesis, we used DNA-damaging reporter strain MDM-623 to assess the interaction between ianthelliformisamines and ciprofloxacin. The data illustrated that ianthelliformisamines did not target DNA gyrase (Figure 5), implying that ciprofloxacin and ianthelliformisamines do not share the same signaling pathway. Thus, their interaction may potentially be synergism rather than additivity.

Ciprofloxacin-induced filamentous growth in all tested strains apart from PAO1Δ*pslA* (Figure 6, Figure 7, Figure 8, Figure 9 and Figure 10). The elongated phenotype was in accordance with the previous study [49]. The treatment of biofilms formed by PAO1 or biofilm matrix component isogenic negative mutants revealed synergistic biofilm-dispersing activity of compounds **1** and **2** each in combination with ciprofloxacin or compound **3** alone targeting the clinically most relevant PDO300, the alginate overproducer (Figure 6, Figure 7, Figure 8, Figure 9 and Figure 10). The treatment biofilms formed knockout mutants incapable of producing one biofilm matrix component such as Psl, Pel, or alginate, suggesting that Psl production mediates resistance to compounds **1**–**3**.

The biofilm inhibition assay here employed LB to grow in vitro biofilms, which possibly share some of the properties of clinical isolates; however, biofilms grown in mucin-rich medium mimicking CF lung sputum would further validate the potency and the efficacy of these combinations. Additionally, the compounds and/or their combinations were not tested on clinical isolates, which are more clinically relevant. We focused on exploring the mechanisms of action of the compounds individually but did not take into account those of the combinations, as the action of one compound could be altered in the presence of another molecule. Though the test compounds showed low toxicity on HEK293 cells, their combination with ciprofloxacin could exert serious undesired effects on human cells. As with all antibiotic treatments, the bactericidal activity of **3** may induce mutagenesis that eventually confers rampant drug resistance. Hence, mutation frequency should be studied to address the concern.

In in vitro metabolism assays of ianthelliformisamines A–C (**1**–**3**), the results of the positive control verapamil were consistent with published data [50] and confirmed suitable activity of the microsomes under the conditions used. In this study, **1** underwent moderate degradation in mouse liver microsomes but displayed significant instability in the presence of human microsomes, while **2** was moderately degraded in microsomes from both species. In contrast, **3** was metabolically stable, with virtually no degradation observed in incubations with liver microsomes from either species. This information may serve as guidance for additional structure optimization to improve potency and avoid metabolic liabilities.

## 4. Materials and Methods

### 4.1. Bacterial Strains, Chemicals and Media

Wild-type *P. aeruginosa* strains PAO1 (prototrophic wild-type) [51] and different isogenic mutant strains including PAO1Δ*pslA* (Δ*pslA*, Psl deletion mutant), PAO1Δ*pelF* (Δ*pelF*, Pel deletion mutant) [41], PDO300 (mucoid, Δ*mucA22*, alginate-overproducing mutant) [52], and PDO300Δ*alg8* (Δ*alg8*, alginate deletion mutant) [53] were used in this study. Bacterial strains were grown in Luria-Bertani (LB) medium (10 g/L tryptone, 10 g/L sodium chloride, and 5 g/L yeast extract) at 37 °C. Ciprofloxacin hydrochloride hydrate was purchased from Alfa Aesar. Meropenem trihydrate, resazurin, polymyxin B, and *N*-phenylnaphthylamine (NPN) were obtained from Aldrich-Sigma, Burlington, MA, USA. 

Ianthelliformisamines A–C (**1**–**3**) were obtained from the Davis Open Access Natural Product-Based Library. The details of the marine sponge material source and the extraction and isolation processes used to purify and identify these marine natural products are described elsewhere [26]. The molecular weights of compounds **1**–**3** are 520.30 g/mol, 463.21 g/mol, and 838.26 g/mol, respectively.

The Davis Open Access Natural Product-Based Library currently consists of 512 distinct compounds, the majority (53%) of which are natural products that have been obtained from Australian natural sources (https://www.griffith.edu.au/institute-drug-discovery/unique-resources/naturebank, accessed on 5 May 2023), such as endophytic fungi [54], plants [55], macrofungi [56], and marine invertebrates [57]. Approximately 28% of this library contains semi-synthetic natural product analogues [58], while a smaller percentage (19%) are known commercial drugs or synthetic compounds inspired by natural products. The Davis Open Access Library is housed within Compounds Australia (www.compoundsaustralia.com) as 5-mM DMSO solutions. Library compounds were either isolated in quantities ranging from 0.2 mg to >50 mg or purchased from commercial suppliers. The natural product isolation procedures or semi-synthetic studies for the majority of compounds in this unique library have been previously published [54,55,56,57]. All compounds were >95% pure when submitted for storage within Compounds Australia.

### 4.2. Determination of Minimum Inhibitory Concentration (MIC)

The minimum inhibitory concentration (MIC) of compounds and antibiotics were performed as previously described, with minor modification [59]. The overnight cultures of *P. aeruginosa* PAO1 at 37 °C and 200 rpm in LB medium were washed once with sterile saline 0.9% (*w*/*v*) and adjusted to an OD_600_ of 0.05, and 1% inoculum was transferred into fresh LB medium. After incubation at 37 °C and 200 rpm for 6 h to reach the mid-log phase, the cells were washed once with sterile saline 0.9% and diluted to an OD_600_ of 0.0004 (*v*/*v*). Three antibiotics, including tobramycin, ciprofloxacin, and meropenem, were dissolved in water and filter sterilized. Ten microliters of two-fold serially diluted antibiotics were transferred to a 96-well Costar plate (Corning Inc., Corning, MA, USA) followed by the addition of 90 µL of diluted cultures. After an 18-h incubation at 37 °C in the static condition, optical density OD_600_ was monitored using a Synergy 2 microplate reader (BioTek, Winooski, VT, USA). The growth control (90 µL of diluted cells and 10 µL of sterile water) and the sterility control/background value (90 µL of LB medium and 10 µL of sterile water) were included in each plate. The MIC was determined as the lowest concentrations of antibiotics that inhibit complete bacterial growth compared to growth control. 

### 4.3. Biofilm Inhibition Assay

The overnight cultures at 37 °C in LB medium were washed once with sterile saline 0.9% (*w*/*v*) and adjusted to an OD_600_ of 0.05, and 1% inoculum was transferred into a fresh LB medium. Following incubation at 37 °C, 200 rpm for 6–6.5 h to reach the mid-log phase, the cells were washed once with sterile saline 0.9% and diluted to an OD_600_ of 0.01. Then, 45 µL aliquots were dispensed into 384-well plates (Greiner Bio-One, Kremsmünster, Austria, catalog no. 781091). In biofilm inhibition assays, test compounds (5 µL) were loaded prior to the addition of bacteria. The plates were incubated for 24 h at 37 °C in static conditions. The effects of compounds on bacterial growth and viability of biofilm bacteria were determined by the OD_600_ and resazurin metabolic assay, respectively. The final concentrations of DMSO in the assays were 1–2%. The negative controls or untreated cultures consisted of inoculum and different concentrations of DMSO. Antibiotic ciprofloxacin (0.25 µg/mL and 25 µg/mL) was used as positive controls. The initial OD_600_ and final OD_600_ were read before incubation at 37 °C and after 24 h incubation, respectively, followed by assessment of biofilm viability by resazurin metabolic assay. The experiments were carried out with three technical replicates and repeated at least twice. The growth inhibition was calculated as a function of the percentage of the total bacterial inhibition using Equation (1), where A_B_ is the OD_600_ of LB medium, and A_T0_ and A_Tf_ are the OD_600_ recorded at inoculating time and at the end of the assay.

(1) $$\%\;\mathrm{Inhibition}=100\;\times \;[\;1\;-\;\left[\frac{{\mathrm{OD}}_{600\;}\left(A_{\mathrm{sample}\;}\mathrm{Tf}-{\;A}_{\mathrm{sample}\;}\;T0\right)-\left(A_{B\;}\mathrm{Tf}\;-{\;A}_{B\;}T0\right)}{{\mathrm{OD}}_{600\;}\left(A_{\mathrm{negative}\;\mathrm{control}\;}\mathrm{Tf}-{\;A}_{\mathrm{negative}\;\mathrm{control}\;}\;T0\right)-\left(A_{B\;}\mathrm{Tf}\;-{\;A}_{B\;}T0\right)}\right]]$$

Resazurin (RSZ) is a non-toxic and non-fluorescent blue compound that is irreversibly reduced by living cells through electron transfer reactions to highly fluorescent resorufin derivatives. RSZ has been extensively used to assess antibacterial and anti-parasite activities and as an indicator for cell viability [60,61]. 

Resazurin sodium salt (Sigma-Aldrich, St. Louis, MI, USA) was dissolved in Milli-Q water at 0.2% (*w*/*v*) and filter sterilized. The solution was stored at −20 °C in the dark. The assay was performed as previously described with modification [62]. The cultures were withdrawn, and the plates were washed three times with sterile water using the plate washer (BioTek). To remove the remaining water in the wells, the plates were tapped with autoclaved paper towels. An amount of 50 µL of diluted RSZ solution in LB medium was added into each well, followed by incubation at 37 °C for 5–6 h. A microplate reader (BioTek Synergy 2) was used to measure the fluorescence intensity (excitation 530 nm, emission 590 nm). Data collected were determined as a function of fluorescence percentage using Equation (2), where F_B_ is the fluorescence of the diluted RSZ solution in LB medium in the absence of *P. aeruginosa* cells.

(2) $$\%\;\mathrm{Inhibition}=[\frac{\left(F_{\mathrm{negative}\;\mathrm{control}\;-}F_{B\;}\right)-\left(F_{\mathrm{sample}\;-}F_{B\;}\right)}{\left(F_{\mathrm{negative}\;\mathrm{control}\;-\;}F_{B\;}\right)}]\times 100$$

### 4.4. Checkerboard Assay

The synergistic activity of compounds with antibiotics, including tobramycin, ciprofloxacin and meropenem, was investigated by a checkerboard dilution assay [63,64]. Briefly, mid-log cultures of *P. aeruginosa* were diluted 1:1250 before being added to the plates. Next, 90 µL aliquots of diluted cells were added to a 96-well Costar (Corning) plate containing either 10 µL of compounds and/or 10 µL of antibiotics. Compounds and antibiotics were tested at 11 and 7 concentrations, respectively. The first row contained antibiotics alone, while the first column contained compounds alone, and the remaining columns were for combinations. Since the compounds did not exhibit significant bactericidal activity apart from compound **3**, the recorded data were not analyzed using the FIC (fractional inhibitory concentration) index. The combinations were considered synergistic if the compounds decreased 3–4 fold in the MIC of antibiotics.

### 4.5. Ethidium Bromide Efflux Assay

To investigate the efflux pump-inhibiting activity of test compounds on wild-type PAO1, an efflux assay using ethidium bromide (EtBr) as a substrate was performed as previously described [65]. An overnight culture of PAO1 in LB broth was incubated in a shaker at 37 °C until it reached an OD_600_ of 0.5. The bacterial cells were washed with phosphate buffered saline (PBS) and centrifuged, and the pellet was resuspended in PBS. Test compounds, 5 µL, were added to the flat-bottomed, black 96-well-plate followed by addition of 90 µL of the bacterial suspension. EtBr at a non-toxic concentration (5 µL, 2 µg/mL) was added before the plates were read. The fluorescence reading was recorded at an excitation of 530 nm and emission of 590 nm every min for 60 min using a BioTek Synergy H1 Hybrid microplate reader. Carbonyl cyanide m-chlorophenyl hydrazone (CCCP), a chemical inhibitor of efflux pumps, was used as the positive control, and DMSO (1–2%) was used as the vehicle control, as compounds were dissolved in DMSO. Untreated cells were used as the negative control.

### 4.6. NPN Uptake Assay

The permeability of the outer membrane upon exposure to test compounds was assessed using a 1-*N*-phenylnapthylamine (NPN) uptake assay [29,66]. An overnight culture of PAO1 was grown to an OD_600_ of 0.5, centrifuged, washed with HEPES buffer (5 mM, pH 7.2), and re-suspended in HEPES buffer containing 100 µM CCCP. The mixture was then incubated in the dark at room temperature for 15 min to inactivate efflux pumps, followed by washing with HEPES buffer. The pellet was resuspended in assay buffer (5 mM HEPES, 5 mM glucose, pH 7.2) supplemented with 10 µM NPN. Ninety microliters of the cell suspension were added to the flat-bottomed, black 96-well plate containing 10 µL of test compounds, and the NPN fluorescence was immediately monitored using a BioTek Synergy H1 Hybrid microplate reader at an excitation of 350 nm and an emission of 420 nm every min for 30 min. Polymyxin B at 6.4 µg/mL and 10 µg/mL was used as the positive control, and DMSO (1–2%) was used as the vehicle control, as compounds were dissolved in DMSO. Untreated cells were used as the negative control.

### 4.7. Cellular Bioluminescent Assay

The reporter strain MDM-623 was kindly provided by Dr. Timothy Opperman (Microbiotix, Inc., Worcester, MA, USA). The strain harbors a promoter region from *PA0614* fused to *Photorhabdus luminescens* luciferase *luxCDABE* operon, which was constructed to respond to DNA damage. All experiments were carried out as previously described with minor modification [33]. The bacteria were grown overnight in an LB medium containing 1 mM IPTG, 20 µg/mL gentamicin, and 12.5 µg/mL tetracycline. Next, overnight cultures were inoculated in an LB medium containing 1 mM IPTG, 20 µg/mL gentamicin, and 12.5 µg/mL tetracycline and grown in a shaker until they reached an OD_600_ of 0.5. The bacteria were centrifuged and washed with 0.9% saline, and then 90 µL of the washed cells were transferred into 96-well plates containing 10 µL of test compounds. Luminescence was monitored using a BioTek Synergy H1 Hybrid microplate reader for 8 h at 30-min intervals. Ciprofloxacin at different concentrations was used as a positive control, and DMSO was also included as the negative control.

### 4.8. Cytotoxicity Assay

Cytotoxicity of the test compounds was assessed on human embryonic kidney (HEK293) cells [67]. Cells were grown and diluted in DMEM: Ham F12 medium supplemented with 10% FBS. Test compounds (10 µL) were added into 384-well plates prior to the transfer of 55 µL of cells at a concentration of 3.2 × 10^5^ cells/mL. The plates were incubated for 24 h at 37 °C followed by the addition of 10 µL of resazurin at a final concentration of 70 µM and further incubation for 5 h at 37 °C. The resazurin fluorescence was read at an excitation of 530 nm and an emission of 595 nm in the BioTek Synergy H1 Hybrid microplate reader.

### 4.9. Fluorescence Microscopy

For biofilm bacteria imaging, bacteria were cultured, treated with test compounds, and washed, as previously mentioned in the biofilm inhibition assay. The biofilm bacteria were stained using a LIVE/DEAD BacLight bacterial viability kit (Molecular Probes, Inc., Eugene, OR, USA), which consists of SYTO9 (green; staining cells with intact membrane) and propidium iodide (red; staining cells with compromised membrane) [41]. Images were captured using a high-content imaging system Operetta CLS (Perkin Elmer, Waltham, MA, USA) with a 60× water-immersion objective.

### 4.10. Stability of Ianthelliformisamines A–C (***1**–**3***) in Mouse and Human Liver Microsomes

#### 4.10.1. In Vitro Incubation

Microsomes were obtained commercially: (1) mouse microsomes (male CD-1; Lot # M5042-C and (2) human microsomes (mixed-sex, 50-donor pool; Lot # PL050E-C) (Gibco, Grand Island, NY, USA). The metabolic assays were performed based on a previously described method [68]. Briefly, each tube (12 × 75 mm glass) contained potassium phosphate buffer (0.1 M, pH 7.4, containing glucose-6-phosphate dehydrogenase (G-6-P-DH; 1 IU/mL), microsomes (0.5 mg/mL), and test compound (**1**, **2** or **3**) or positive control (verapamil [VPL]), 1.0 μM. Reactions were initiated by the addition of 25 μL of a 20× stock of an NADPH regeneration system (NRS) containing NADP, glucose-6-phosphate (G-6-P), and MgCl_2_, to provide final concentrations of 1.3 mM, 3.5 mM, and 3.3 mM, respectively. Total incubation volume was 0.5 mL. Metabolic stability of **1**, **2**, or **3** was determined in triplicate in an oscillating water bath (Grant) at 37 °C and 100 RPM. Positive control (verapamil, 1.0 μM) and negative control (**1**, **2**, or **3** without NRS) incubations were conducted concurrently in singlicate. Stock solutions of the test compounds (5.0 or 10.0 mM) were prepared in DMSO, and spiking solutions of these, as well as verapamil, were prepared in acetonitrile at 100 µM. The final organic (ACN) concentration in the incubation tubes was 1%. Tubes containing buffer, microsomes, and test (or control) compound (5 mM) were preincubated at 37 °C for 5 min prior to the reaction being initiated by the addition of NRS. The negative control was initiated by the addition of the test compound. Samples (50 μL) were removed at t = 0 (immediately after reaction start), 5, 10, 20, 30, and 60 min. Samples were added to microcentrifuge tubes containing 150 μL of ice-cold acetonitrile containing internal standard (clotrimazole; for **1** = 10.0 ng/mL + 0.5% (*v*/*v*) formic acid; for **1** and **2** = 2.0 ng/mL + 0.5% (*v*/*v*) formic acid; for VPL = 20.0 ng/mL + 0.1% (*v*/*v*) formic acid). Tubes were centrifuged (13,000× *g*, 5 min), and ~135 μL of supernatant transferred to polypropylene 96-well plates for analysis.

#### 4.10.2. Analysis

Samples were analyzed by HPLC-MS/MS (Waters 2795 solvent delivery system linked to a MicroMass Quatromicro tandem MS/MS). Chromatographic separation was achieved using a biphenyl column [Kinetix, 100 mm × 4.6 mm, 2.6 μm; Phenomenex (Sydney, Australia)], coupled with a gradient elution with mobile phases containing acetonitrile/H_2_O/0.5% formic acid. Analysis was conducted in positive ion multiple reaction monitoring (MRM) mode with argon as the collision gas. Optimum MS/MS conditions were established prior to incubations by direct infusion of individual solutions of **1**, **2**, and **3** into the LC-MS/MS. To ensure adequate sensitivity of analysis, multiple mass transitions were summed for all three test compounds. The mass transitions monitored for **1** were *m*/*z* 521.4 > 447.5 + 521.4 > 375.7 amu, for **2** were *m*/*z* 464.3 > 375.7 + 464.3 > 318.9 amu, and for **3** were *m*/*z* 839.7 > 447.3 + 839.7 > 375.5 amu, while for VPL and CTZ, single transitions were monitored (VPL: *m*/*z* 455.3 > 164.9; CTZ: *m*/*z* 277.1 > 165.1 amu). Linearity of LC-MS/MS analysis of the three test compounds in buffer was confirmed over a suitable concentration range prior to incubations being undertaken.

#### 4.10.3. Calculations

Peak area ratios (**1**, **2**, or **3** or VPL: clotrimazole [internal standard]) were determined for each timepoint for each tube and expressed as % remaining (normalized to t = 0 as 100%). LN % remaining vs. time data for each data set were fitted to an exponential decay function to determine the first-order rate constant (k) for substrate depletion. Where possible, the rate of depletion for each sample was used to calculate additional values, as shown in Table 1. For Equations (5)–(7), scaling parameters used were described elsewhere [69].

### 4.11. Statistical Analysis

All experiments were performed in triplicate. The data are the means and standard deviations of two independent experiments. In cytotoxicity assay, non-parametric one-way ANOVA followed by Dunnett’s multiple comparison post-hoc test was performed to compare treatments with the negative control (GraphPad Prism 8.0, GraphPad Software, Inc., San Diego, CA, USA).

## 5. Conclusions

We demonstrated that ianthelliformisamines A–C (**1**–**3**) showed bactericidal activity and prevented *P. aeruginosa* biofilm formation with low cytotoxicity. Compounds **1** and **2** synergistically interacted with ciprofloxacin to influence bacterial growth and mitigate biofilm formation while **3** solely targeted both growth modes, which were also dependent on the ability to produce various exopolymeric substances. These findings further emphasize the crucial roles of exopolysaccharides constituting the biofilm matrix in antibiotic recalcitrance, and they might shed light on the development of novel targets to counteract this superbug. Although the detailed mechanisms of action of these compounds and their combinations remain to be determined, compound **3** mode of action studies showed that it is an effective efflux pump inhibitor. Of the three compounds, **3** showed greatest metabolic stability in the presence of mouse and human liver microsomes. Collectively, we suggest ianthelliformisamines as promising drug leads for future investigations into development treatments for *P. aeruginosa* infections.

## Figures and Tables

**Table 1:** Additional parameters were calculated using listed equations.

Parameter	Unit	Equation Used	
Half life	min	t_½_ = $\frac{\ln\;(2)\;}{k}$	(3)
Cl_int, in vitro_	μL/min/mg protein	k × V where V = incubation volume (μL)/microsomal protein (mg)	(4)
Cl_int_	mL/min/kg	Cl_int, in vitro_ × $\frac{\mathrm{liver}\;\mathrm{mass}\;\left(g\right)}{\mathrm{body}\;\mathrm{weight}\;(\mathrm{kg})}$ × $\frac{\mathrm{microsomal}\;\mathrm{protein}\;(\mathrm{mg})}{\mathrm{liver}\;\mathrm{mass}\;(g)}$	(5)
Cl_blood_	mL/min/kg	$\frac{Q\;\times \;{\mathrm{Cl}}_{\mathrm{int}}}{Q\;+\;{\mathrm{Cl}}_{\mathrm{int}}}$ where Q = hepatic blood flow (mL/min/kg body weight)	(6)
E_H_^1,2^	---	$\frac{{\mathrm{Cl}}_{\mathrm{blood}}}{Q}$ = $\frac{{\mathrm{Cl}}_{\mathrm{int}}}{Q\;+\;{\mathrm{Cl}}_{\mathrm{int}}}\;$	(7)

^1^ Hepatic extraction rate is based on microsomal degradation of each test compound in vitro and assumes that NADPH-dependent oxidative metabolism is the dominant metabolic route. No allowance was made for other Phase 1 or Phase 2 metabolic pathways. ^2^ Microsomal binding of ianthelliformisamines A, B, or C (**1**, **2** or **3**) was not determined. It was assumed that the fraction unbound (fu) = 1.

## Data Availability

The data presented in this study are available on reasonable request from the corresponding author.

## Supplementary Materials

The supporting information can be downloaded at: https://www.mdpi.com/article/10.3390/ijms241210204/s1.
