# Supplementary material for: Bromotyrosine-Derived Metabolites from a Marine Sponge Inhibit Pseudomonas aeruginosa Biofilms
Source: Int J Mol Sci. 2023 Jun 16;24(12):10204. doi: 10.3390/ijms241210204 (PMC10299588; doi:10.3390/ijms241210204)

SUPPORTING INFORMATION

Bromotyrosine-Derived Metabolites from a Marine Sponge Inhibit *Pseudomonas aeruginosa* Biofilms

Tam M. T. Tran [1], Russell S. Addison [2], Rohan A. Davis [3,4], Bernd H. A. Rehm [1,5,*]

[1] Centre for Cell Factories and Biopolymers, Griffith Institute for Drug Discovery, Griffith University, Nathan, QLD 4111, Australia

[2] Preclinical ADME/PK, Griffith Institute for Drug Discovery, Griffith University, Nathan, QLD 4111, Australia

[3] NatureBank, Griffith Institute for Drug Discovery, Griffith University, Nathan, QLD 4111, Australia

[4] School of Environment and Science, Griffith University, Nathan, QLD 4111, Australia

[5] Menzies Health Institute Queensland, Griffith University, Gold Coast, QLD 4222, Australia

[*] correspondence: b.rehm@griffith.edu.au

Table S1. The degradation of ianthelliformisamine A in mouse liver microsomes.

Table S2. Metabolic stability parameters for ianthelliformisamine A in mouse liver microsomes.

Table S3. The degradation of ianthelliformisamine B in mouse and human liver microsomes.

Table S4. Metabolic stability parameters for ianthelliformisamine B in mouse and human liver microsomes.

Table S5. The degradation of ianthelliformisamine C in mouse and human liver microsomes.

Figure S1. Metabolic stability parameters for ianthelliformisamine A in mouse liver microsomes.

Figure S2. Metabolic stability of ianthelliformisamine B in mouse and human liver microsomes.

Figure S3. Metabolic stability of ianthelliformisamine C in mouse and human liver microsomes.

Table S1. The results for the degradation of ianthelliformisamine A (**1**) normalized to t = 0) in mouse liver microsomes

Time (min)	% Remaining	
	Mouse	
	Mean	SD
0	100.00	0.00
5	93.32	14.29
10	96.74	27.23
20	66.26	19.36
30	57.28	20.01
60	53.42	17.07

Table S2. Metabolic stability parameters for ianthelliformisamine A (**1**) based on NADPH-dependent degradation profiles in mouse liver microsomes.

Parameter	Units	Result
k[1]	min^{-1}	0.0218 ± 0.0125
Half life[1]	min	38.2 ± 17.2
$Cl_{int,\ in\ vitro}$	μL/min/mg protein	43.6
Cl_{int}	mL/min/kg	112.5
Cl_{blood}	mL/min/kg	58.1
Hepatic Extraction (E_H) [2]	---	0.48

[1] mean ± SD, n = 3 [2] based on mean data for k

Table S3. The results for the degradation of ianthelliformisamine B (**2**) (normalized to t = 0) in mouse and human liver microsomes.

Time (min)	% Remaining			
	Mouse		Human	
	Mean	SD	Mean	SD
0	100.00	0	100.00	0
5	153.98	14.66	112.15	16.61
10	160.70	65.50	98.42	9.88
20	138.91	39.12	80.61	3.04
30	119.60	41.51	68.41	7.28
60	122.66	35.50	76.42	16.88

Table S4. Metabolic stability parameters for ianthelliformisamine B (**2**) based on NADPH-dependent degradation profiles in mouse and human liver microsomes.

Parameter	Units	Mouse	Human
k[1]	min^{-1}	0.0165 ± 0.0030	0.0195 ± 0.0011
Half life[1]	min	42.8 ± 7.6	35.7 ± 2.1
$Cl_{int,\ in\ vitro}$	µL/min/mg protein	33.0	39.0
Cl_{int}	mL/min/kg	85.1	32.1
Cl_{blood}	mL/min/kg	49.8	12.6
Hepatic Extraction (E_H)[2]	---	0.42	0.61

[1] mean ± SD, n = 3 [2] based on mean data for k

Table S5. The results for the degradation of ianthelliformisamine C (**3**) normalized to t = 0) in mouse and human liver microsomes.

Time (min)	% Remaining			
	Mouse		Human	
	Mean	SD	Mean	SD
0	100.00	0	100.00	0
5	102.69	7.394	105.22	7.357
10	107.54	14.67	102.09	11.38
20	106.48	7.882	104.88	18.772
30	107.42	7.931	101.07	10.566
60	95.80	13.574	99.19	3.752

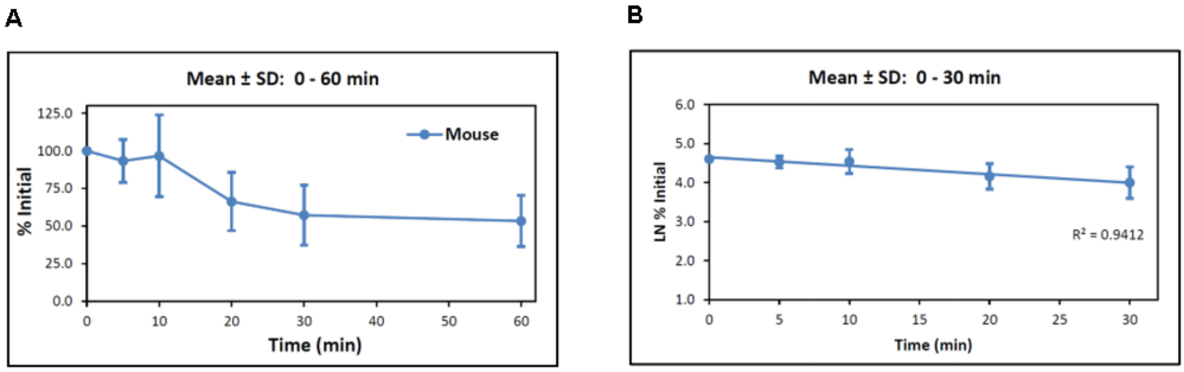

Figure S1. (**A**) Metabolic stability parameters for ianthelliformisamine A (**1)** based on NADPH-dependent degradation profiles in mouse liver microsomes. (**B**) Metabolic stability of **1** (mean ± SD; n = 3; 0 – 30 min) in mouse liver microsomes: LN-transformed data.

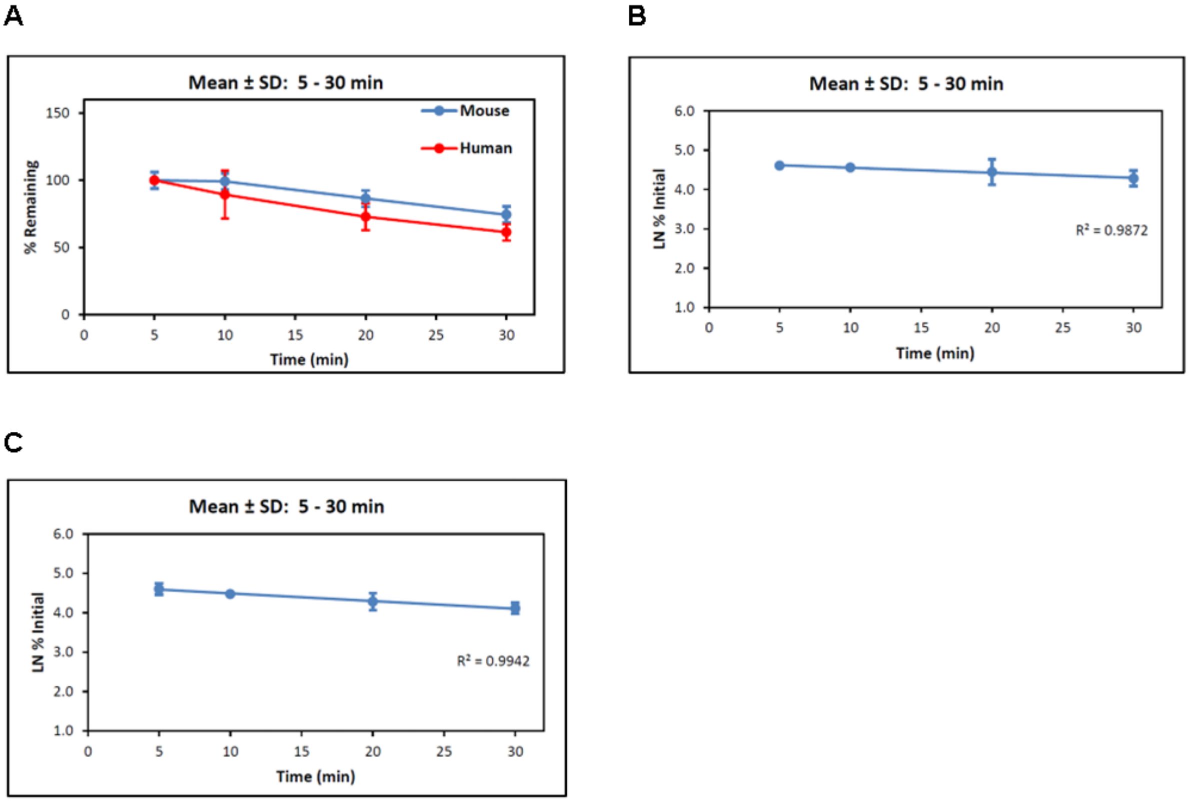

Figure S2. (**A**) Metabolic stability of ianthelliformisamine B (**2)** (mean ± SD; n = 3) in mouse and human liver microsomes. (**B**) Metabolic stability of **2** (mean ± SD; n = 3) in mouse liver microsomes: LN-transformed data (normalized to t = 5 min). (**C**) Metabolic stability of 2 (mean ± SD; n = 3) in human liver microsomes: LN-transformed data (normalized to t = 5 min).

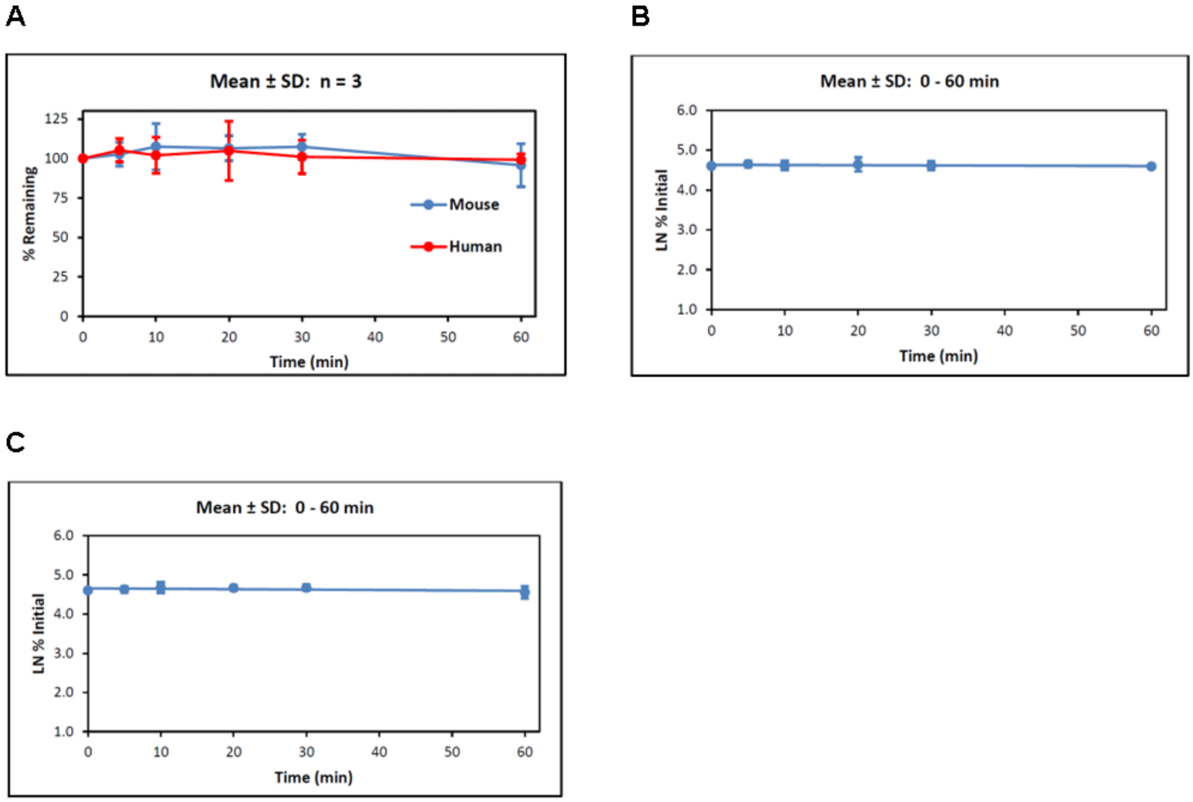

Figure S3. (**A**) Metabolic stability of ianthelliformisamine C (**3)** (mean ± SD; n = 3) in mouse and human liver microsomes. (**B**) Metabolic stability of **3** (mean ± SD; n = 3) in human liver microsomes: LN-transformed data. (**C**) Metabolic stability of **3** (mean ± SD; n = 3) in mouse liver microsomes: LN-transformed data.

Supplementary Data

Ianthelliformisamine A

- **Mouse liver microsomes**

Ianthelliformisamine A **Mouse Liver Microsomes**

Lot # MS042-C

	Tube 1			Tube 2			Tube 3			Mean % Initial			
Time (min)	PAR	% Initial	LN % Initial	PAR	% Initial	LN % Initial	PAR	% Initial	LN % Initial	Mean	SD	Mean LN	SD
0	0.936	100	4.605	1.023	100	4.605	1.42	100	4.605	100.00	0.00	4.605	0.000
5	0.857	91.6	4.517	1.109	108.4	4.686	1.136	80.0	4.382	93.32	14.29	4.528	0.152
10	0.983	105.0	4.654	1.216	118.9	4.778	0.942	66.3	4.195	96.74	27.23	4.542	0.307
20	0.754	80.56	4.389	0.757	74.00	4.304	0.628	44.2	3.789	66.26	19.36	4.161	0.324
30	0.632	67.52	4.212	0.717	70.09	4.250	0.486	34.23	3.533	57.28	20.01	3.998	0.403
60	0.613	65.49	4.182	0.423	41.35	3.722				53.42	17.07	3.952	0.325
	t half (min)	(0-30 min)	52.5		(0-30 min)	43.0		(0-30 min)	19.2	Mean t half (0-30 min)		38.2	17.2
	k (min^{-1})	(0-30 min)	0.0132		(0-30 min)	0.0161		(0-30 min)	0.0361	Mean k (0-30 min)		0.0218	0.0125
	t half (min)	(0-60 min)	87.7		(0-60 min)	99.0		(0-60 min)		Mean t half (0-60 min)		93.4	8.0
	k (min^{-1})	(0-60 min)	0.0079		(0-60 min)	0.0168		(0-60 min)		Mean k (0-60 min)		0.0124	0.0021

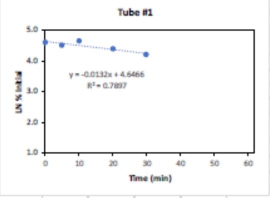

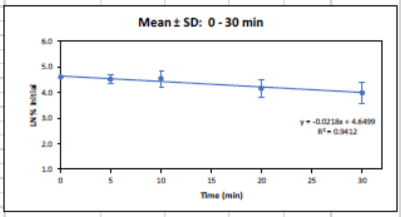

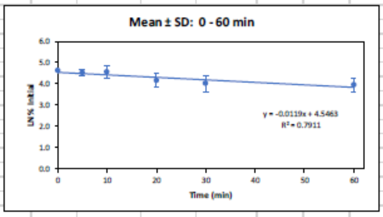

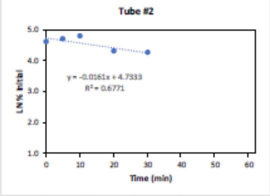

t half (0-30 min) **31.8** min

t half (0-60 min) **56.8** min

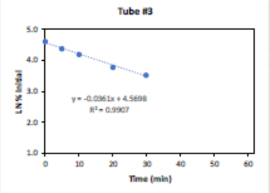

	Negative Control		
Time (min)	PAR	% Initial	LN % Initial
0	1.003	100.00	4.605
5	1.318	131.41	4.878
10	1.324	132.00	4.883
20	1.349	134.50	4.902
30	0.861	85.84	4.453
60	0.673	67.10	4.206
t half (0-30 min)	113.6	min	
k (min^{-1})	0.0061	min $^{-1}$	
t half (20-60 min)	45.3	min	
k (min^{-1})	0.0153	min $^{-1}$	

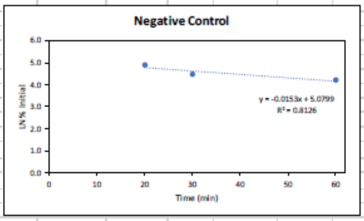

	VPL Control		
Time (min)	PAR	% Initial	LN % Initial
0	16.266	100.00	4.605
5	7.348	45.17	3.811
10	3.083	18.95	2.942
20	1.369	8.42	2.130
30	0.592	3.64	1.292
60	0.157	0.97	-0.035

t half (0-30 min) 6.5 min

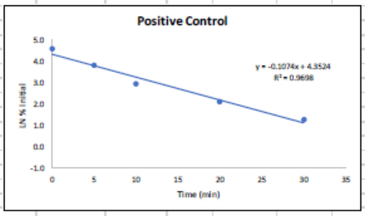

Ianthelliformisamine B

- **Mouse liver microsomes**

IANTH B | **Mouse Liver Microsomes**

Lot # MS042-C

	Tube 1			Tube 2			Tube 3			Mean % Initial			
Time (min)	PAR	% Initial	LN % Initial	PAR	% Initial	LN % Initial	PAR	% Initial	LN % Initial	Mean	SD	Mean LN	SD
0	1.280	100	4.605	0.778	100	4.605	1.184	100	4.605	100.00	0	4.605	0.000
5	2.117	100.0	4.605	1.322	100.0	4.605	1.688	100.0	4.605	100.00	0.000	4.605	0.000
10	1.885	89.0	4.489	1.804	136.5	4.916	1.219	72.2	4.280	99.24	33.314	4.562	0.324
20	1.641	77.5	4.350	1.418	107.3	4.675	1.258	74.5	4.311	86.43	18.099	4.446	0.200
30	1.271	60.0	4.095	1.302	98.5	4.590	1.091	64.6	4.169	74.39	20.998	4.285	0.267
60	1.650	77.9	4.356	1.203	91.0	4.511	1.000	59.2	4.082	76.06	15.962	4.316	0.217
	t half (min)	(5-30 min)	35.4		(10-30 min)	42.5		(5-30 min)	50.6	Mean t half (5-30 min)		42.8	7.6
	k (min^{-1})	(5-30 min)	0.0196		(10-30 min)	0.0163		(5-30 min)	0.0137	Mean k (5-30 min)		0.0165	0.0030

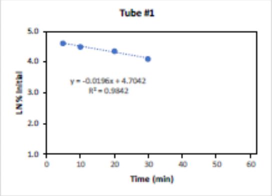

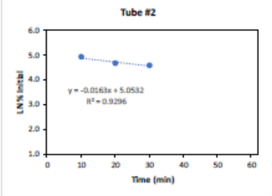

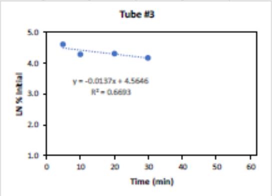

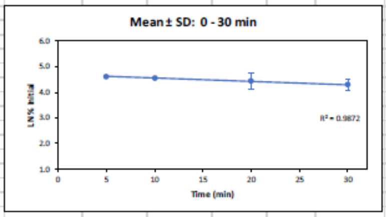

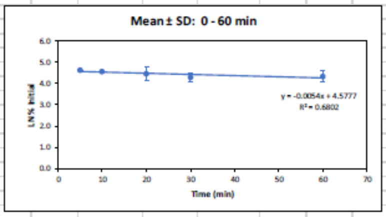

t half (5-30 min) **41.5** min

t half (0-60 min) **128.0** min

	Negative Control		
Time (min)	PAR	% Initial	LN % Initial
0	1.273	100.00	4.605
5			
10	1.509	118.54	4.775
20	1.340	105.26	4.656
30	1.294	101.65	4.622
60	1.259	98.90	4.594
t half (10-30 min)	90.0	min	
t half (10-60 min)	231.0	min	

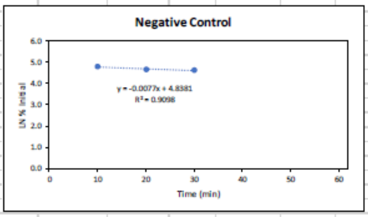

	VPL Control		
Time (min)	PAR	% Initial	LN % Initial
0	9.091	100.00	4.605
5	3.816	41.98	3.737
10	2.115	23.26	3.147
20	0.849	9.34	2.234
30	0.353	3.88	1.357
60	0.115	1.26	0.235
t half (0-20 min)	6.0	min	
t half (0-30 min)	6.6	min	

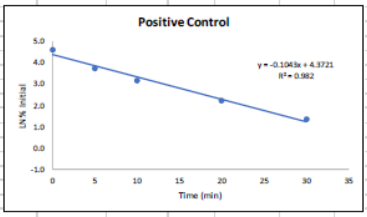

- **Human liver microsomes**

IANTH B	Human Liver Microsomes													
	Lot #	PL050E-C												

	Tube 1			Tube 2			Tube 3			Mean % Initial			
Time (min)	PAR	% Initial	LN % Initial	PAR	% Initial	LN % Initial	PAR	% Initial	LN % Initial	Mean	SD	Mean LN	SD
0	1.676	76.4	4.335	2.088	94.4	4.547	1.709	100.5	4.610	90.41	12.54	4.498	0.144
5	2.195	100.0	4.605	2.212	100.0	4.605	1.701	100.0	4.605	100.00	0.000	4.605	0.000
10	1.519	69.2	4.237	2.287	103.4	4.639	1.625	95.5	4.559	89.38	17.91	4.478	0.213
20	1.378	62.8	4.140	1.61	72.8	4.288	1.410	82.9	4.418	72.82	10.06	4.282	0.139
30	1.227	55.9	4.024	1.503	67.9	4.219	1.026	60.3	4.100	61.39	6.095	4.114	0.098
60	1.568	71.4	4.269	1.249	56.5	4.034	1.297	76.2	4.334	68.05	10.32	4.212	0.158
	t half (min)	(5-30 min)	34.3		(5-30 min)	38.1		(5-30 min)	34.7	Mean t half (5-30 min)		35.7	2.1
	k (min^{-1})	(5-30 min)	0.0202		(5-30 min)	0.0182		(5-30 min)	0.0200	Mean k (5-30 min)		0.0195	0.0011

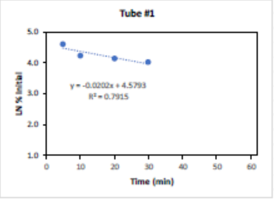

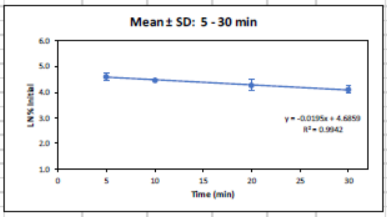

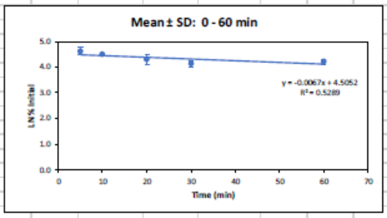

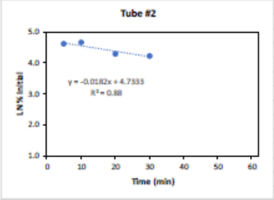

t half (5-30 min) 35.5 min

t half (0-60 min) 103.4 min

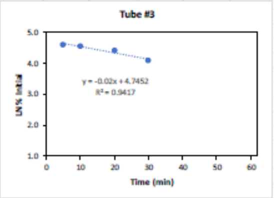

	Negative Control		
Time (min)	PAR	% Initial	LN % Initial
0	1.120	100.00	4.605
5	1.413	126.16	4.838
10	1.752	156.43	5.053
20	1.482	132.32	4.885
30	1.286	114.82	4.743
60	1.175	104.91	4.653
t half (5-30 min)	103.4	min	
t half (5-60 min)	126.0	min	

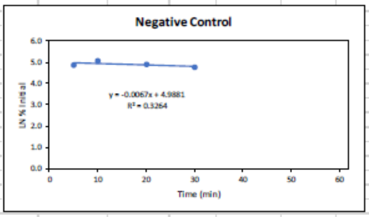

	VPL Control		
Time (min)	PAR	% Initial	LN % Initial
0	11.067	100.00	4.605
5	10.718	96.85	4.573
10	8.039	72.64	4.286
20	4.651	42.03	3.738
30	3.128	28.26	3.342
60	1.559	14.09	2.645
t half (0-30 min)	15.3	min	

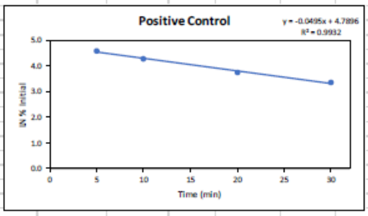

Ianthelliformisamine C

- **Mouse liver microsomes**

IANTH C | Mouse Liver Microsomes

Lot # MS042-C

	Tube 1			Tube 2			Tube 3			Mean % Initial			
Time (min)	PAR	% Initial	LN % Initial	PAR	% Initial	LN % Initial	PAR	% Initial	LN % Initial	Mean	SD	Mean LN	SD
0	15.525	100	4.605	13.057	100	4.605	15.779	100	4.605	100.00	0	4.605	0.000
5	15.402	99.2	4.597	14.517	111.2	4.711	15.413	97.7	4.582	102.69	7.394	4.630	0.071
10	14.797	95.3	4.557	16.165	123.8	4.819	16.334	103.5	4.640	107.54	14.67	4.672	0.134
20	15.122	97.40	4.579	14.565	111.55	4.714	17.436	110.5	4.705	106.48	7.882	4.666	0.076
30	15.620	100.61	4.611	15.163	116.13	4.755	16.651	105.53	4.659	107.42	7.931	4.675	0.073
60	12.536	80.75	4.391	13.985	107.11	4.674	15.708	99.55	4.601	95.80	13.574	4.555	0.147
	t half (min)	(0-30 min)	3465.0		(0-30 min)	223.5		(0-30 min)	239.0	Mean t half (0-30 min)		1309.2	1867.0
	k (min⁻¹)	(0-30 min)	0.0002		(0-30 min)	0.0031		(0-30 min)	0.0029	Mean k (0-30 min)		0.0021	0.0016
	t half (min)	(0-60 min)	216.6		(0-60 min)	77000.0		(0-60 min)	6930.0	Mean t half (0-60 min)		28048.9	42525.6
	k (min⁻¹)	(0-60 min)	0.0032		(0-60 min)	0.000009		(0-60 min)	0.0001	Mean k (0-60 min)		0.0011	0.0016

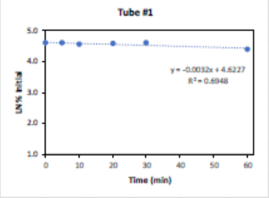

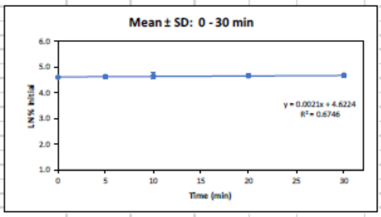

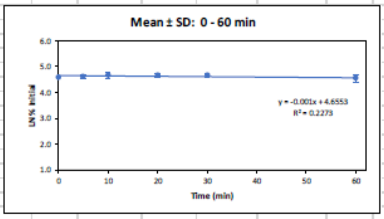

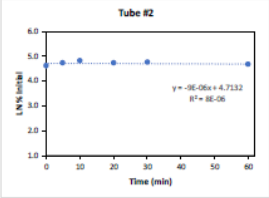

t half (0-30 min) 330.0 min (+VE)

t half (0-60 min) 693.0 min

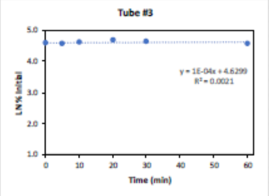

	Negative Control		
Time (min)	PAR	% Initial	LN % Initial
0	17.5	100.00	4.605
5	15.543	88.82	4.487
10	12.204	69.74	4.245
20	13.459	76.91	4.343
30	15.755	90.03	4.500
60	17.908	102.33	4.628
t half (0-30 min)	256.7	min	
t half (0-60 min)	277.2	min (+ve)	

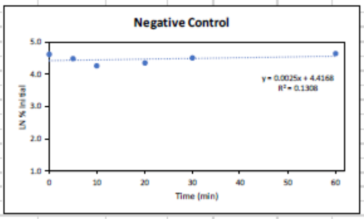

	VPL Control		
Time (min)	PAR	% Initial	LN % Initial
0	7.703	100.00	4.605
5	4.296	55.77	4.021
10	1.858	24.12	3.183
20	0.746	9.68	2.271
30	0.476	6.18	1.821
60	0.145	1.88	0.633
t half (0-20 min)	5.9	min	
t half (0-30 min)	7.3	min	

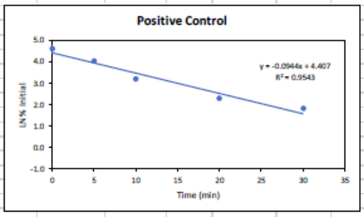

- ## Human liver microsomes

IANTH C — Human Liver Microsomes

Lot # PL050E-C

	Tube 1			Tube 2			Tube 3			Mean % Initial			
Time (min)	PAR	% Initial	LN % Initial	PAR	% Initial	LN % Initial	PAR	% Initial	LN % Initial	Mean	SD	Mean LN	SD
0	17.071	100	4.605	19.446	100	4.605	18.466	100	4.605	100.00	0	4.605	0.000
5	18.441	108.0	4.682	18.839	96.9	4.573	20.455	110.8	4.707	105.22	7.357	4.654	0.071
10	17.332	101.5	4.620	17.694	91.0	4.511	21.003	113.7	4.734	102.09	11.38	4.622	0.112
20	16.717	97.93	4.584	17.613	90.57	4.506	23.292	126.1	4.837	104.88	18.772	4.643	0.173
30	17.663	103.47	4.639	17.406	89.51	4.494	20.355	110.23	4.703	101.07	10.566	4.612	0.107
60	17.219	100.87	4.614	18.453	94.89	4.553	18.801	101.81	4.623	99.19	3.752	4.597	0.038
	t half (min)	(0-30 min)	1732.5		(0-30 min)	198.0		(5-30 min)	192.5	Mean t half (5-30 min)		707.7	887.5
	k (min^{-1})	(0-30 min)	0.0004		(0-30 min)	0.0035		(5-30 min)	0.0036	Mean k (5-30 min)		0.0025	0.0018
	t half (min)	(0-60 min)	2310.0		(0-60 min)	1155.0		(5-60 min)	990.0	Mean t half (5-60 min)		1485.0	719.2
	k (min^{-1})	(0-60 min)	0.0003		(0-60 min)	0.0006		(5-60 min)	0.0007	Mean k (5-60 min)		0.0005	0.0021

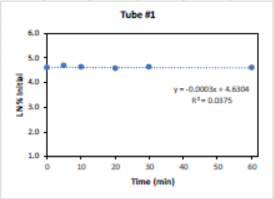

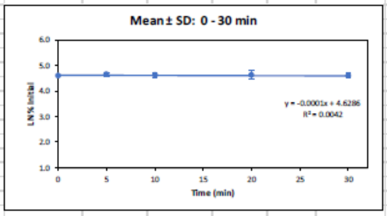

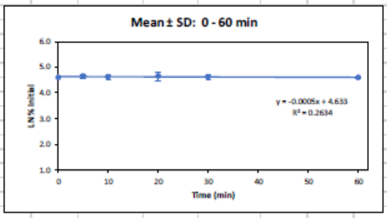

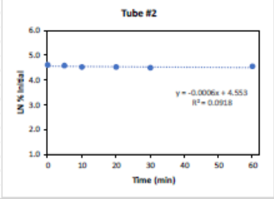

t half (0-30 min) 6930.0 min

t half (0-60 min) 1386.0 min

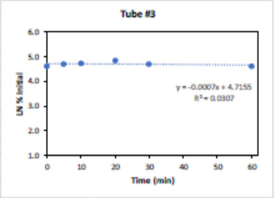

	Negative Control		
Time (min)	PAR	% Initial	LN % Initial
0	17.852	100.00	4.605
5	18.737	104.96	4.654
10	18.634	104.38	4.648
20	17.489	97.97	4.585
30	18.295	102.48	4.630
60	21.781	122.01	4.804
t half (0-30 min)	1732.5	min	
t half (0-60 min)	247.5	min (+VE)	

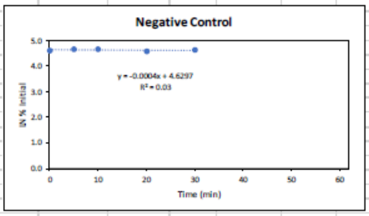

	VPL Control		
Time (min)	PAR	% Initial	LN % Initial
0	9.686	100.00	4.605
5	8.626	89.06	4.489
10	6.559	67.72	4.215
20	4.16	42.95	3.760
30	2.817	29.08	3.370
60	1.441	14.88	2.700
t half (0-60 min)	21.2	min	
t half (0-30 min)	16.2	min	

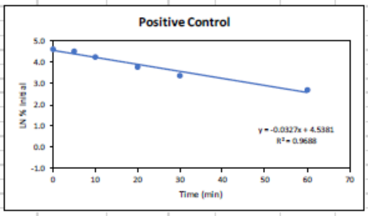

Supplement: Supplementary file 1 [file ijms-24-10204-s001.zip › ijms-2416803-supplementary.pdf]
